# On the effectiveness of limited-data large language model fine-tuning for Arabic

**DOI:** 10.1371/journal.pone.0332419

**Published:** 2025-10-08

**Authors:** Mohamed Alkaoud

**Affiliations:** Department of Computer Science, College of Computer and Information Sciences, King Saud University, Riyadh, Saudi Arabia; Philadelphia University, JORDAN

## Abstract

This paper presents an investigation into fine-tuning large language models (LLMs) for Arabic natural language processing (NLP) tasks. Although recent multilingual LLMs have made remarkable progress in zero-shot and few-shot settings, specialized models such as fine-tuned BERT variants continue to define state-of-the-art (SOTA) performance in many Arabic tasks. We demonstrate that by fine-tuning a general-purpose LLM (GPT-4o mini) on only a small subset (3.0%–7.5%) of the training samples, we exceed previous best reported results in sentiment analysis (ArSAS) and sarcasm detection (ArSarcasm), while achieving performance statistically comparable to the SOTA in news categorization (ASND). This study highlights that LLMs, when properly adapted, can outperform established models without relying on full-scale annotated training sets. Furthermore, our analysis with the open-source Gemma-3-27B model confirms the generalizability of our data-efficient method. Notably, this approach enabled the model to achieve performance statistically comparable to SOTA on all three tasks, although the proprietary GPT-4o mini maintained an overall performance advantage. We further compare GPT-4o with GPT-4o mini to examine the impact of model size on fine-tuning. GPT-4o outperforms GPT-4o mini across all sample sizes but by small margins (<1%). Notably, GPT-4o fine-tuned on 100 samples achieves marginally better performance than GPT-4o mini fine-tuned on 500 samples, indicating that larger models require fewer labeled examples. Additionally, we find that fine-tuning performance follows predictable scaling, with GPT-4o mini’s performance growth function accurately estimating GPT-4o’s results (error < 0.005). This enables efficient performance estimation for larger models using smaller ones. Our findings emphasize the practical benefits of fine-tuning LLMs for Arabic NLP, while demonstrating predictable scaling laws that can guide efficient model selection and adaptation.

## 1 Introduction

The landscape of NLP has shifted rapidly with the emergence of transformer-based [1] models. While initial gains were observed through models such as BERT [2], RoBERTa [3], and their variants, the subsequent phase of progress has come from very large models, LLMs, pre-trained on extensive multilingual corpora. These LLMs have shown remarkable capabilities in tasks that were once considered challenging, and they have often achieved this through zero-shot and few-shot learning methods. Yet, many specialized tasks, especially in Arabic, still rely on fine-tuned versions of non-LLM models to achieve SOTA performance [4].

Arabic is an example of a language where general-purpose large language models often do not reach SOTA performance [4]. Current SOTA models have used Arabic-specific models fine-tuned on large annotated datasets. While these models achieve high accuracy, the reliance on extensive training sets remains a practical concern. Generating large annotated corpora is often time-consuming and costly.

This paper examines the following two questions: 1) whether an LLM that is not specialized solely in Arabic can be fine-tuned to outperform current SOTA models, and 2) how much fine-tuning data is needed for an LLM to reach SOTA level. We focus in this work on social media–related tasks because the language used on these platforms often differs from more formal written Arabic. Social media posts can contain dialectal words, emojis, and informal expressions that pose unique challenges for standard NLP pipelines. By addressing these settings, we aim to test how well our fine-tuning approach adapts to spontaneous and often unpredictable linguistic features. Additionally, social media data is abundant and carries practical value in tasks such as sentiment tracking, topic classification, and sarcasm detection. However, labeling for social media content can be costly because words change meaning over time [5]. Moreover, the language and trends in social media shift at a rapid pace. As an illustration, the MNIST [6] dataset was released in 1998 and remains useful to this day, since handwriting styles for digits have changed very little. In contrast, social media text from even a year ago might already be out of date, given that new slang, memes, and cultural references appear continuously. This fluidity makes it necessary to refresh or expand labeled datasets frequently, driving up the time and resources required for maintaining high-quality annotations.

By focusing on three social media related tasks: 1) sentiment analysis, 2) news categorization, and 3) sarcasm detection; we test the generality of our approach. Our results show that a very small portion of the training data is sufficient to surpass the best current models. Our contributions can be summarized as:

1. We show that fine-tuning a large multilingual language model (GPT-4o mini) on a small subset of Arabic training data (as little as 0.6% of the dataset) can surpass the performance of SOTA models that rely on extensive fine-tuning of Arabic-specific models.
2. We achieve SOTA results on three distinct Arabic social media NLP tasks—sentiment analysis (ArSAS), news categorization (ASND), and sarcasm detection (ArSarcasm).
3. We analyze the relationship between fine-tuning dataset size and model performance, showing that while performance improves with more data, the most significant gains occur with relatively small datasets.
4. We formalize the notion of performance scaling and notice that GPT-4o follows a very similar growth function to GPT-4o mini on ArSAS.

We structure the paper as follows. Section 2 presents background information on and reviews related work. Section 3 describes our methodology, including the datasets, fine-tuning process, and experimental setup. Section 4 presents the main results and compares them to existing baselines; additionally, it provides an analysis of the results. Section 5 concludes with key insights and recommendations for continued research.

## 2 Background and related work

Historically, Arabic NLP progressed from rule-based systems and early statistical methods to neural architectures and pretrained encoders. The shift to transformer-based models brought performance boosts. Models such as AraBERT [7], CAMeLBERT [8], and ARBERT [9] adapted the BERT [2] architecture to Arabic. These models served as strong baselines for many Arabic NLP tasks. However, despite improvements, these systems generally required full-scale training data to reach optimal performance.

In parallel, the global NLP community has produced multiple LLMs [10–13] trained on massive amounts of textual data. These models often include Arabic text in their training sets. Although they can handle Arabic to some extent, their performance on specialized tasks lags behind localized models that have been fine-tuned extensively [4]. Their main advantage lies in flexibility—they can switch between tasks and languages, and they often show acceptable zero- or few-shot performance. The question we explore here is whether fine-tuning these LLMs on small subsets of Arabic training sets can bridge or even surpass the performance gap relative to fine-tuned Arabic-specific models.

The relative performance of LLMs and non-LLM models varies depending on the specific NLP task and language. Several studies directly compare their capabilities. In the domain of information extraction from emergency department notes, Choi et al. [14]. found that a fine-tuned Llama-2 [15] model outperformed various BERT models in extracting information related to injury mechanism, place of occurrence, activity, intent, and severity. The Llama-2 model achieved accuracies ranging from 0.774 to 0.972 across these tasks, exceeding the performance of BERT models. Similarly, in the context of customer review classification and sentiment analysis, Roumeliotis et al. [16] demonstrated that GPT-4o (without fine-tuning) outperformed BERT, achieving an F1-score of 65.5% compared to BERT’s 60.8%. They also showed that fine-tuning both GPT-4o and GPT-4o mini doesn’t improve performance by much.

In a study on detecting suicidal ideation, Oliveira et al. [17] found that while Bing/GPT-4 achieved the highest accuracy (98%) among LLMs, fine-tuned BERTimbau-Large models [18] achieved a comparable accuracy of 96%. In the context of standardizing obstetric diagnostic terminology, Wang et al. [19] found that a BERT-based model [20] performed better than LLMs (ChatGLM2 and Qwen-14B-Chat). Similarly, a study by Bucher et al. [21] showed that fine-tuned small models such as BERT consistently outperformed large language models in various text classification tasks. In addition to that, Godoy et al. [22] found that GPT-4 achieved an F1-score of 0.80 in zero-shot learning and 0.96 in five-shot learning for named entity recognition (NER) in mammography radiology reports, comparable to a specialized NER-BERT model’s F1-score of 0.97 beating both zero and few-shot LLMs. In classifying patient intent during telephone consultations, Cho et al. [23] found that GPT-4 achieved an accuracy of 85.2% (in a few-shot setting), significantly outperforming BERT (71.3%) and LSTM [24] (73.7%), particularly in handling ambiguous queries.

While LLMs have shown potential for Arabic language processing, their performance compared to existing SOTA models requires further investigation. A key study in this area is the LAraBench benchmark, presented by Abdelali et al. [2], which offers the first known comprehensive evaluation of LLM performance for Arabic across a wide range of NLP and speech processing tasks. This benchmark covers 33 tasks across 61 datasets. Abdelali et al. findings indicate that while LLMs demonstrate promising results, consistently outperforming random baselines, they often fall short of SOTA models in various tasks. This performance gap highlights the need for further research to improve the effectiveness of LLMs for Arabic, particularly in areas such as model fine-tuning.

Our contribution lies in showing that targeted fine-tuning of a large multilingual model on a subset of Arabic training data can exceed performance levels that were previously achieved only by fully fine-tuned, Arabic-specific models.

## 3 Approach

We begin with a large multilingual model that has been pretrained on a wide range of languages, including Arabic (GPT-4o mini). Rather than relying on zero-shot or few-shot inference directly, we fine-tune the model on a relatively small portion of the available training data.

### 3.1 Tasks and datasets

**Fig 1:**
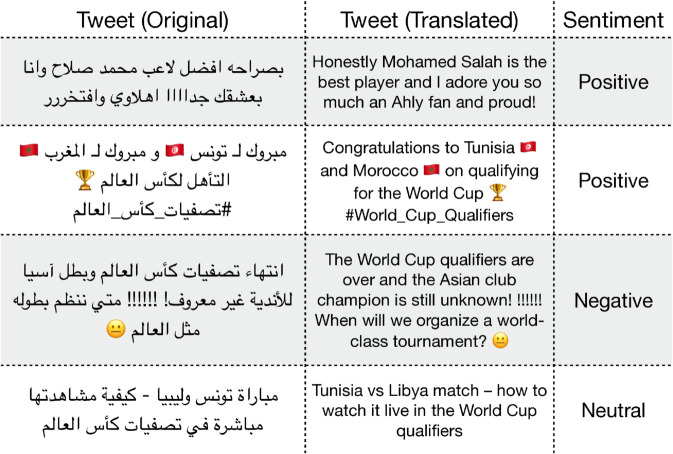
Examples from the ArSAS dataset. The *Tweet (Original)* column displays the original Arabic tweet, while the *Tweet (Translated)* column provides its English translation. The *Sentiment* column indicates the sentiment expressed in each tweet. Note that some tweets include dialectal Arabic, and translations may not fully convey the intended meaning.

**Fig 2:**
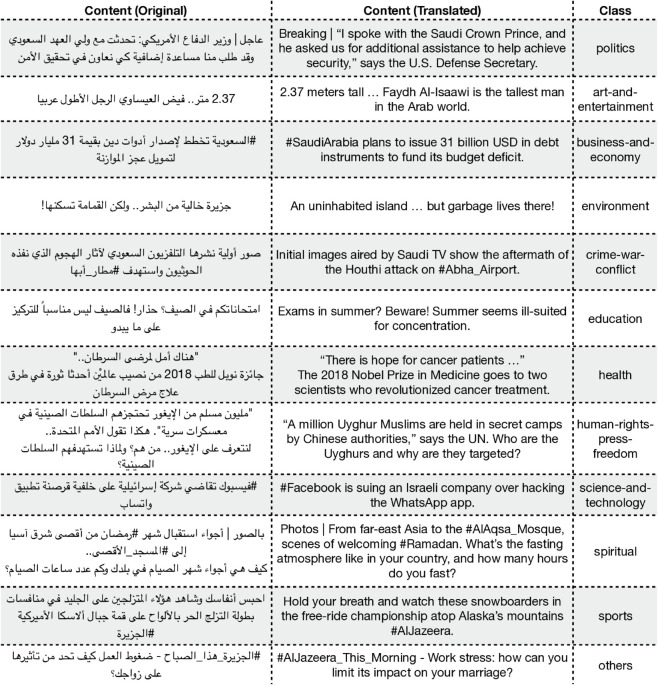
Examples from the ASND dataset. The *Content (Original)* column displays the original Arabic news post, while the *Content (Translated)* column provides its English translation. The *Class* column indicates the classification of the news post.

**Fig 3:**
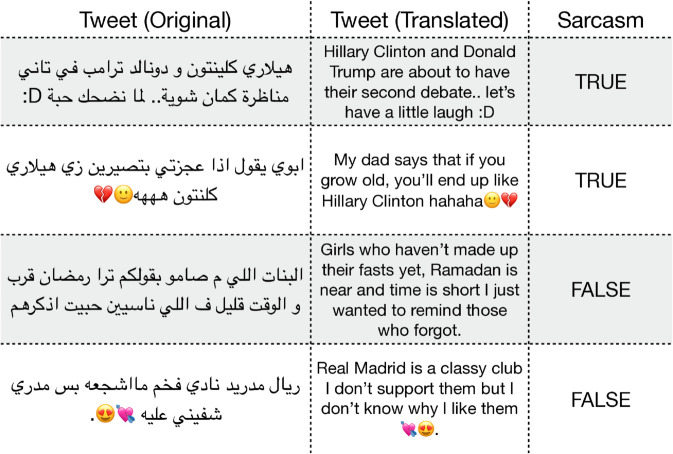
Examples from the ArSarcasm dataset. The *Tweet (Original)* column displays the original Arabic tweet, while the *Tweet (Translated)* column provides its English translation. The *Sarcasm* column indicates whether the tweet is sarcastic or not. Note that some tweets include dialectal Arabic, and translations may not fully convey the intended meaning.

We focus on three tasks that reflect different aspects of Arabic social media NLP tasks:

1. Sentiment Analysis (ArSAS [25]): A dataset of Arabic tweets annotated for sentiment (positive, negative, neutral, mixed). This task involves handling informal language and dialectal words, as well as emoji and other non-standard textual elements. Examples from the dataset are shown in Fig 1.
2. News Categorization (ASND [26]): A dataset for Arabic social media news categorization. Posts require classification into topical categories, testing the model’s ability to capture thematic content in short texts. Examples from the dataset are shown in Fig 2.
3. Sarcasm Detection (ArSarcasm [27]): A dataset designed for sarcasm detection in Arabic social media text. Sarcasm can be subtle, requiring an understanding of pragmatic cues and the interaction between literal meaning and implied intent. Examples from the dataset are shown in Fig 3.

These three datasets were chosen because they represent critical application areas in Arabic social media analysis and have well-established baselines and evaluation metrics. They were also chosen because Abdelali et al. [4] showed that their zero-shot LLM performance is always lower than existing non-LLM models. Table 1 shows statistics about the three datasets. For ArSAS [25], we combine the ‘neutral’ and ‘mixed’ classes like it was done in the current SOTA model [28] that we compare our performance to. We don’t change the training set labels, we only change the classes (by merging ‘neutral’ and ‘mixed’) during inference time when evaluating performance.

**Table 1:** The number of training and test set examples for ArSAS, ASND, and ArSarcasm.

Dataset	# of Training Set Examples	# of Test Set Examples
ArSAS	16,851	4,213
ASND	6,676	1,103
ArSarcasm	8,437	2,110

### 3.2 Fine-tuning

We fine-tune GPT-4o mini (gpt-4o-mini-2024-07-18) using the OpenAI fine-tuning API. We choose GPT-4o mini due to its balance of speed, cost-effectiveness, and suitability for large-scale experiments. For each of the three tasks, we create two distinct fine-tuning configurations: one uses 100 training points and another uses 500 training points. In each case, we build a validation set of the same size (100 and 500 samples, respectively). The subsets for our experiments were generated using a two-step procedure to ensure a random partition of data for both training and validation. Initially, we performed random sampling on the full training dataset to draw sets of 200 and 1,000 samples for the 100-sample and 500-sample configurations, respectively. This process was implemented using the pandas.DataFrame.sample() function, with a fixed random state (42) to ensure reproducibility. Subsequently, each of these pools was evenly partitioned into two halves, yielding the final training and validation sets (e.g., 100 samples for training and 100 for validation). We apply the automated hyperparameter settings provided by OpenAI’s fine-tuning service, which determines values based on internal heuristics. Concretely, this setup yields three training epochs, a batch size of one, and a learning rate multiplier of 1.8 across all tasks and configurations. Although more extensive experimentation with hyperparameters might improve results further, we are limited by budget constraints and thus rely on these default parameters. The seeds were randomly generated by OpenAI’s fine-tuning service.

In our investigation of model scaling behavior, we conducted a series of systematic experiments with GPT-4o mini using the ArSAS dataset. We structured the experiments to reveal how the model’s performance evolves with increasing amounts of training data, starting from just 50 samples and progressively scaling up through multiple orders of magnitude: 500 samples, 1,000 samples, half the dataset (8,425 samples), and finally the complete dataset of 16,851 samples.

Given our focus on understanding the relationship between dataset size and model performance, we made the deliberate choice to allocate all available labeled data for training. While this meant foregoing a validation set, it allowed us to explore the full extent of potential performance gains from additional training data.

We conducted our scaling experiments on ArSAS because it offers the largest sample size among our datasets, with 16,851 examples. This substantial dataset size makes ArSAS particularly well-suited for studying how model performance evolves across different data scales. By starting with just 50 samples and progressively scaling up to the full dataset, we could trace a more complete learning curve that spans multiple orders of magnitude. The other datasets (ASND and ArSarcasm) would have limited our ability to observe long-range scaling behavior, potentially missing important trends that only become apparent with larger sample sizes. The extensive size of ArSAS allowed us to investigate both the early-stage rapid improvements with limited data and any potential plateaus or diminishing returns that might emerge at larger scales. For the hyperparameters of each expirement, we also use the automated OpenAI hyperparameters selector giving us the following values: Three training epochs, a batch size of one, and a learning rate multiplier of 1.8 for the 50 and 500 samples. For the 1000 sample, we get three epochs, a batch size of two, and a learning rate multiplier of 1.8. For the 8,425 samples, we get one epoch, a batch size of five, and a learning rate multiplier of 1.8. Finally, for the 16,851 samples, we have one epoch, a batch size of eleven, and a learning rate multiplier of 1.8.

The experiments were executed using the LLMeBench framework [29], a framework specifically developed to support the structured and reproducible evaluation of large language models. LLMeBench integrates key components such as data loading, prompt generation, and performance assessment into a unified workflow. This design minimizes potential configuration inconsistencies and enhances the reliability of cross-run comparisons, thereby ensuring methodological rigor in the evaluation process.

To provide a comprehensive analysis and contextualize the performance of the proprietary GPT-4o mini, we extended our investigation to include an open-source alternative. Our model of choice is gemma-3-27b-it [30], selected on the basis that it was the top-performing open-source LLM in its size class (10B to 35B parameters) on the Arabic Broad Benchmark (ABB) [31,32] as of June 9, 2025. For the fine-tuning process of Gemma, we used Low-Rank Adaptation (LoRA) [33].

### 3.3 Baselines

We compare against existing literature that reports SOTA results on ArSAS [28], ASND [26], and ArSarcasm [4].

In our evaluation, we use the established SOTA score of 0.758 [28] for the ArSAS dataset. It is important to distinguish this from other reported results, such as the “Mazajak” system by Farha and Magdy [34]. While their work is significant, their results are not directly comparable to ours due to critical differences in the experimental setup. Specifically, the “Mazajak” study modifies the task by removing the “mixed” sentiment class and further curates the data by only including tweets with a confidence level above 50%. As the data splits used in their study are also not reported, a direct like-for-like comparison is not feasible. To perform our due diligence, we evaluated ArSAS’s test set, taken from LLMeBench, using the publicly available “Mazajak” online platform (Available at: http://mazajak.inf.ed.ac.uk:8000/). This test yielded a macro-F1 score of 0.738, which is lower than Hassan et al.’s [28] macro-F1 score of 0.758 that we compare against. Therefore, given these significant differences in data and methodology, we maintain that the 0.758 score is the most appropriate and rigorous SOTA benchmark for our study.

### 3.4 Prompts

To ensure consistency and comparability between the fine-tuning and inference phases, identical prompts were utilized throughout the experimental pipeline. This strategy minimizes variability stemming from differences in prompt design, thereby isolating the impact of model fine-tuning on performance. The prompts were structured with specific roles for each component: the **system prompt** provided the instructional context, the **user prompt** contained the tweet or social media post, and the **assistant prompt** returned the predicted label. Below are the system prompts designed for each task:

- **ArSAS:** *Choose only one sentiment between: Positive, Negative, Neutral, or Mixed for each user input.*
- **ASND:** *You are a news categorizing assistant. You will classify what you receive into one of these classes: politics, art-and-entertainment, human-rights-press-freedom, crime-war-conflict, sports, environment, health, science-and-technology, business-and-economy, spiritual, education, or others.*
- **ArSarcasm:** *You are an expert in social media sarcasm detection in Arabic. Predict whether the following tweet is sarcastic or not. Return "True" if the tweet is sarcastic and "False" if it is not.*

We use the same prompts for Gemma, but we append “Only return the classification and nothing else.” to the first two prompts since we noticed Gemma kept returning classes not mentioned in the prompt. For the third task, no modification was made.

### 3.5 Infrastructure

The fine-tuning process for the GPT-4o family was conducted on the cloud using OpenAI’s fine-tuning service. For the inference phase, we run the LLMeBench framework on an Apple M1 MacBook Air equipped with 16GB of unified memory and running macOS Monterey. The Python version used was 3.9.12. For Gemma, fine-tuning was performed using the Unsloth library [35], a Python library optimized for memory-efficient training of large models, on an NVIDIA A100 GPU rented via the vast.ai platform.

### 3.6 Evaluation metrics

For sentiment analysis and news categorization, we use macro-F1 to measure the model’s performance. The F1 score for each class *j* is given by:

$$F_{j}=\frac{2P_{j}R_{j}}{P_{j}+R_{j}},$$ (1)

with *P*_*j*_ and *R*_*j*_ representing the precision and recall for class *j*, respectively. The macro-F1 score is then computed as:

$$\text{Macro-F1}=\frac{1}{K}\sum_{j=1}^{K}F_{j}.$$ (2)

where *K* denotes the total number of classes in the classification task. For sarcasm detection, F1-score (of the positive label) is used:

$$\text{F1}=\frac{2PR}{P+R},$$ (3)

where *P* and *R* denote the precision and recall for the positive class. The main reason we use these specific metrics is because these are the metrics used for the evaluation of the three datasets in current SOTA models [4].

## 4 Results and discussion

### 4.1 Fine-tuning on limited data

Table 2 shows the performance of the fine-tuned GPT-4o mini on three tasks: sentiment analysis, news categorization, and sarcasm detection. As mentioned in the previous section, for each task, two fine-tuning configurations were tested using 100 and 500 training samples. Table 3 details the fine-tuning datasets used for the experiments, including the total number of samples available for each task and the percentage of the dataset utilized for fine-tuning.

**Table 2:** Performance of fine-tuned GPT-4o mini on various tasks compared to previous SOTA. For sentiment analysis and news categorization, the metric used is macro-F1. For sarcasm detection, F1-score is used. ‘Ft.’ in the table refers to fine-tuned.

Task	Dataset	Pre. SOTA	Ft. (100 samples)	Ft. (500 samples)
Sentiment Analysis	ArSAS	0.758 [28]	0.778	**0.781**
News Categorization	ASND	0.770 [26]	0.713	**0.781**
Sarcasm Detection	ArSarcasm	0.504 [4]	0.353	**0.578**

**Table 3:** Fine-tuning data details for GPT-4o mini on the three datasets. The last column (*n*/*N*) shows the percentage of the number of fine-tuning samples over the total number of samples in the training data. ‘ft.’ in the table refers to fine-tuned.

Dataset	# of samples used for ft. (*n*)	Total # of samples in the training data (*N*)	*n*/*N* (%)
ArSAS	100	16,851	0.6%
	500	16,851	3.0%
ASND	100	6,676	1.5%
	500	6,676	7.5%
ArSarcasm	100	8,437	1.2%
	500	8,437	5.9%

The results demonstrate a clear advantage for the 500-sample configuration across all tasks. In sentiment analysis using the ArSAS dataset, even the 100-sample configuration achieved a macro-F1 score of 0.778, surpassing the previous SOTA of 0.758 [28] which is based on fine-tuning BERT. When increased to 500 samples, the performance further improved to 0.781. This improvement is particularly noteworthy considering that these results were achieved using just 0.6% and 3.0% of the available training data, respectively. For news categorization using the ASND dataset, we observe an interesting progression. While the 100-sample configuration (0.713) fell short of the previous SOTA (0.770) [26] which utilized a transformer-based BERT model called QARiB, increasing the training samples to 500 yielded a macro-F1 score of 0.781. This improvement was achieved while using only 7.5% of the available training data, highlighting the effective learning capabilities of LLMs with only a small amount of data. For ArSarcasm, the performance after fine-tuning for 100 samples is the worst among all the tasks in our experiments only achieving an F1-score of 0.353. We attribute this to the difficulty of the task where only a hundred samples may not have been enough. Increasing the fine-tuning data to 500 samples results in a substantial performance gain, achieving an F1-score of 0.578. This result surpasses the previous SOTA of 0.504, which was obtained using GPT-4 in a three-shot setting.

As we can see in Table 3, for both configurations (100 and 500 samples), only a small fraction of the total dataset was used, with the highest percentage being 7.5% for the news categorization task with 500 samples. Table 3 provides insights into how little data was actually used for fine-tuning. For instance, the sarcasm detection task was fine-tuned on just 5.9% of the available dataset (500 out of 8,437 samples), yet it still produced results that improved upon the previous SOTA (an F1-score jump from 0.504 to 0.578). These outcomes show the efficiency of LLMs in extracting meaningful patterns from small-scale data, a feature that is especially valuable in scenarios where extensive annotation is not feasible or cost-effective.

To validate the promising initial results from the 500-sample configuration and to ensure their statistical robustness, we repeated the fine-tuning process four times for each task, using different random seeds for each run, giving us a total of five runs if we include the original run. This approach allows us to account for performance variability and provide a more reliable assessment of the model’s capabilities. The aggregated results of these validation runs are presented in Table 4, which includes the mean, standard deviation, and 95% confidence interval for each task.

**Table 4:** Performance of fine-tuned GPT-4o mini (500 samples) across five runs. Metrics include mean, standard deviation (SD), and 95% confidence interval (CI) using the t-distribution. For sentiment analysis and news categorization, the metric is macro-F1. For sarcasm detection, F1-score is used.

Task	Dataset	Pre. SOTA	Mean ± SD (95% CI)
Sentiment Analysis	ArSAS	0.758 [28]	0.779 ± 0.004 (CI: [0.774, 0.783])
News Categorization	ASND	0.770 [26]	0.776 ± 0.009 (CI: [0.765, 0.787])
Sarcasm Detection	ArSarcasm	0.504 [4]	0.571 ± 0.025 (CI: [0.540, 0.602])

The findings from this validation process solidify our initial conclusions. For both sentiment analysis and sarcasm detection, the fine-tuned GPT-4o mini establishes a new, statistically significant SOTA. In sentiment analysis, the model achieved a mean macro-F1 of 0.779, with a tight 95% confidence interval of [0.774, 0.783] that lies entirely above the previous SOTA of 0.758. The advance is even more pronounced in sarcasm detection, where the model’s mean F1-score of 0.571 represents a substantial leap forward. The corresponding confidence interval of [0.540, 0.602] confirms that this improvement is highly significant. For news categorization, while the mean score of 0.776 is numerically superior to the SOTA, the 95% confidence interval [0.765, 0.787] contains the previous SOTA value of 0.770, indicating that the performance is statistically comparable. Overall, these multi-run experiments confirm that fine-tuning GPT-4o mini on a limited dataset is an effective and reliable strategy for achieving and, in several cases, significantly exceeding SOTA performance in Arabic NLP tasks.

#### 4.1.1 Error analysis.

The following qualitative error analysis was conducted on the predictions from our initial 500-sample fine-tuning experiments for each of the three datasets, with the single-run performance metrics shown in Table 2. For each task, we analyzed a random sample of misclassified examples to identify common patterns of failure and better understand the model’s behavior beyond quantitative metrics.

**Fig 4:**
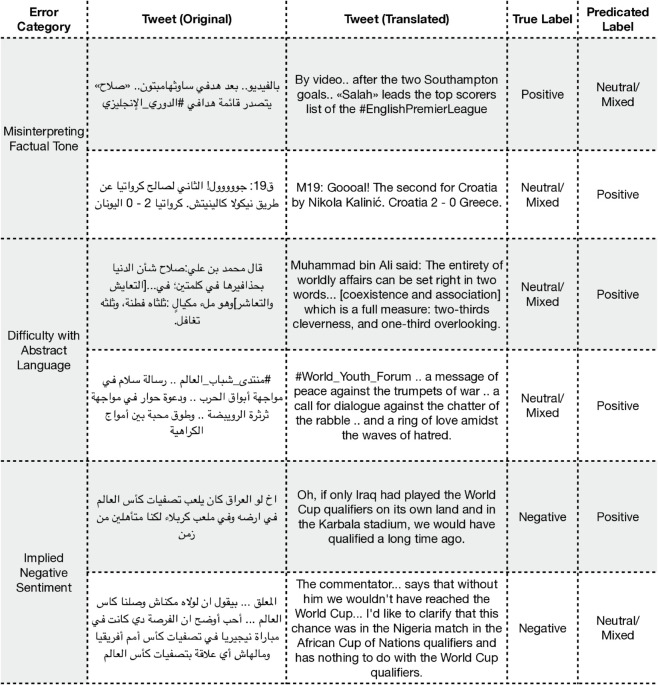
Examples of misclassified predictions from the ArSAS dataset. Note that some tweets include dialectal Arabic, and translations may not fully convey the intended meaning.

**ArSAS: Sentiment analysis.** For the ArSAS dataset, our analysis revealed that the model’s primary weaknesses involve interpreting journalistic tone and implied sentiment. We identified three main error categories, with representative examples presented in Fig 4. The first pattern involves misinterpreting the neutral tone of factual reporting, where the model confuses the sentiment of an event with the tone of its reporting. The second cluster shows difficulty with abstract or philosophical language, where the model defaults to a positive classification based on keywords, missing the neutral context. Finally, the model struggles to grasp implied negative sentiment, such as regret or critique, when it is not conveyed by explicit negative words. It is crucial to note that the “Misinterpreting Factual Tone” error category highlights an inherent subjectivity in sentiment annotation. For instance, a factual news report about a positive event (e.g., a player scoring a goal) can be reasonably labeled as either positive (reflecting the event’s sentiment) or neutral (reflecting the text’s objective tone). This ambiguity presents a significant challenge for any model, as it must learn from potentially inconsistent signals in the training data. Therefore, some of the model’s errors in this category may represent a plausible alternative interpretation of the text, rather than a clear failure of linguistic understanding.

**Fig 5:**
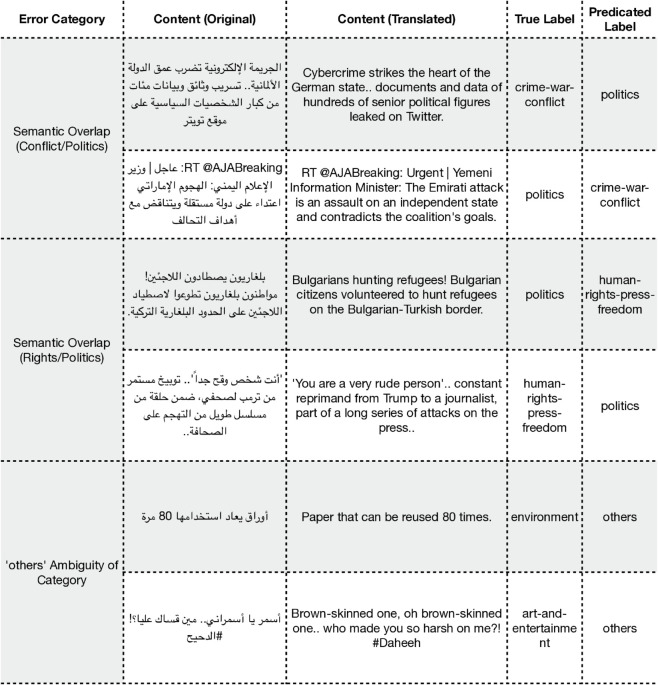
Examples of misclassified predictions for the ASND dataset. Note that some tweets include dialectal Arabic, and translations may not fully convey the intended meaning.

**ASND: News categorization.** For the ASND dataset, the analysis shows that the model’s primary challenge lies in resolving the semantic overlap between closely related news categories as shown in Fig 5. The most frequent confusions occur between ‘politics‘ and ‘crime-war-conflict‘, and between ‘politics‘ and ‘human-rights-press-freedom‘. In these cases, the model often correctly identifies the broad theme but fails to discern the text’s primary focus. Furthermore, the model shows difficulty with the ‘others‘ category, sometimes failing to classify distinct topics like ‘environment‘ or ‘art-and-entertainment‘ correctly.

**Fig 6:**
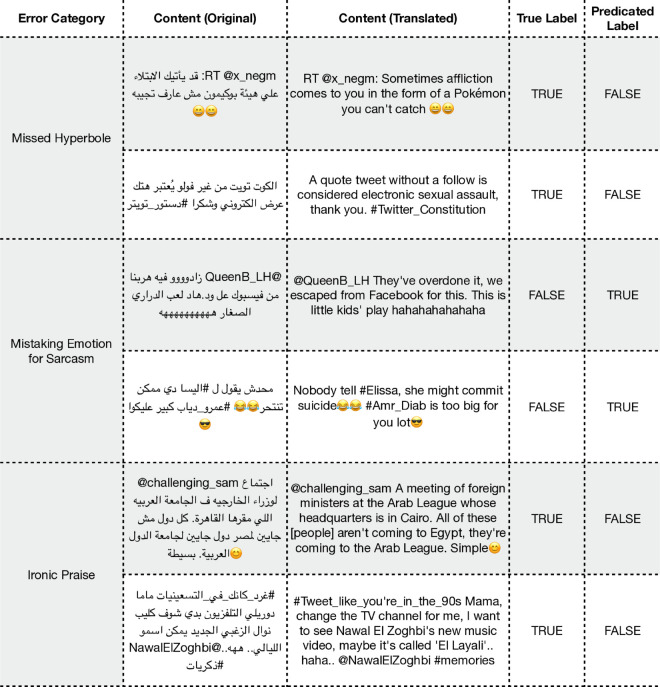
Examples of misclassified predictions for the ArSarcasm dataset. Note that some tweets include dialectal Arabic, and translations may not fully convey the intended meaning.

**ArSarcasm: Sarcasm detection.** The error analysis for the ArSarcasm dataset highlights the profound challenge of distinguishing literal from non-literal language, a task that requires deep pragmatic and contextual understanding. Our analysis of the misclassified examples revealed three primary error categories, with representative examples shown in Fig 6. The first common type of error involves missed hyperbole and absurdity. The model often fails to detect sarcasm when it is conveyed through absurd exaggerations or ridiculous comparisons, such as equating a minor digital annoyance with a major real-world event. It tends to interpret these non-literal statements at face value, missing the mocking or humorous intent. The second category is the opposite problem: mistaking strong emotion for sarcasm. The model exhibits a tendency to classify direct jokes, complaints, or statements with intense emotional language as sarcastic, failing to recognize that such language can also be used sincerely and directly. The third cluster involves a more subtle failure in understanding rhetorical context. This occurs when a text uses a positive or helpful framing to deliver a critical or mocking message. The model successfully reads the positive surface sentiment but completely misses the contextual cues, such as a condescending tone or an undercutting compliment, that invert the statement’s meaning into sarcasm.

### 4.2 Fine-tuning an open-source model on limited data

To provide a comprehensive analysis and contrast the performance of proprietary models with accessible, open-source alternatives, we extended our experiments to include Gemma 3-27b. As mentioned in the previous section, this model was specifically chosen as it represents one of the best-performing open-source models for Arabic with under 35 billion parameters. For the Gemma model, we focused our analysis exclusively on the 500-sample fine-tuning configuration. Since the central thesis of our paper is to demonstrate the effectiveness of limited-data fine-tuning, this specific configuration represents the most relevant and informative point of comparison. It allows for a direct assessment of how a powerful open-source model performs under the same data-efficient conditions where GPT-4o mini already achieved SOTA results. Crucially, to ensure statistical robustness and account for performance variability, each fine-tuning experiment was repeated ten times using different random seeds. The aggregated results, including the mean, standard deviation (SD), and 95% confidence interval (CI) based on a t-distribution, are presented in Table 5. For the experiments utilizing the OpenAI API, we performed five runs per configuration. We determined this number was sufficient to establish a stable performance estimate given the limitations of the OpenAI API. For the open-source Gemma model, fine-tuning was conducted on a cloud GPU instance, which allowed for the flexibility to conduct ten runs per configuration for an even more robust statistical estimate.

**Table 5:** Performance of fine-tuned Gemma (500 samples) across ten runs. Metrics include mean, standard deviation (SD), and 95% confidence interval (CI) using the t-distribution. For sentiment analysis and news categorization, the metric is macro-F1. For sarcasm detection, F1-score is used.

Task	Dataset	Pre. SOTA	Mean ± SD (95% CI)
Sentiment Analysis	ArSAS	0.758 [28]	0.756 ± 0.004 (CI: [0.753, 0.759])
News Categorization	ASND	0.770 [26]	0.772 ± 0.011 (CI: [0.764, 0.780])
Sarcasm Detection	ArSarcasm	0.504 [4]	0.473 ± 0.046 (CI: [0.440, 0.506])

The results from fine-tuning Gemma, summarized in Table 5. For news categorization on the ASND dataset, Gemma achieved a mean macro-F1 score of 0.772, numerically surpassing the previous SOTA of 0.770. However, with the SOTA value falling within the 95% confidence interval of [0.764, 0.780], this result establishes Gemma’s performance as statistically comparable and highly competitive, though not significantly superior.

In sentiment analysis (ArSAS), Gemma’s performance was remarkably consistent, as shown by the very low standard deviation (0.004), achieving a mean macro-F1 of 0.756. This score is only marginally below the SOTA of 0.758, indicating its reliability for this task given that is been trained on only 3% of the training set. For the sarcasm detection task (ArSarcasm), Gemma’s F1-score of 0.473 did not reach the SOTA of 0.504. The wide confidence interval [0.440, 0.506], which contains the SOTA value, suggests that while the mean is lower, the performance is not statistically different. The relatively high standard deviation (0.046) in this task also highlights its inherent difficulty, leading to greater performance variability.

Overall, while Gemma did not outperform the proprietary GPT-4o mini, these findings are significant. They demonstrate that a medium-sized open-source model can achieve performance that is statistically on par with established SOTA benchmarks on multiple tasks with a limited number of labeled data.

### 4.3 Fine-tuning sample size vs. performance

Table 6 provides a detailed analysis of the sentiment analysis task (ArSAS) with varying training sample sizes. As we can notice, the performance improved consistently as the number of training samples increased, starting at 0.703 with zero samples (no fine-tuning) and reaching 0.812 when fine-tuned on the full dataset (16,851 samples). This progression demonstrates the scalability of the model’s performance with increasing data, while achieving 0.7% less compared to the performance of full-dataset with just half of the training data (8,425 samples, achieving 0.803). What this shows is that LLMs can surpass existing SOTA results using a minute amount of data, there are still increases in performance as we increase the size of the dataset. This progression also shows that adding more data generally yields higher performance, but the gains do not scale linearly. The jump from 50 to 500 samples is notable, increasing from 0.736 to 0.778, but subsequent increments (e.g., 500 to 1,000 samples) show diminishing returns per additional sample. Nonetheless, the model still benefits from additional data, surpassing 0.80 performance with only half of the dataset and ultimately reaching 0.812 with the full dataset.

**Table 6:** Performance of GPT-4o mini on ArSAS with varying fine-tuning sample sizes. The last column (*n*/*N*) shows the percentage of the number of fine-tuning samples over the total number of samples in the training data.

Sample Size	macro-F1	*n*/*N* (%)
0 (no fine-tuning)	0.703	0.00 %
50	0.736	0.30%
500	0.778	2.97%
1,000	0.790	5.94%
8,425 (50%)	0.803	50.00%
16,851 (100%)	0.812	100.00%

**Fig 7:**
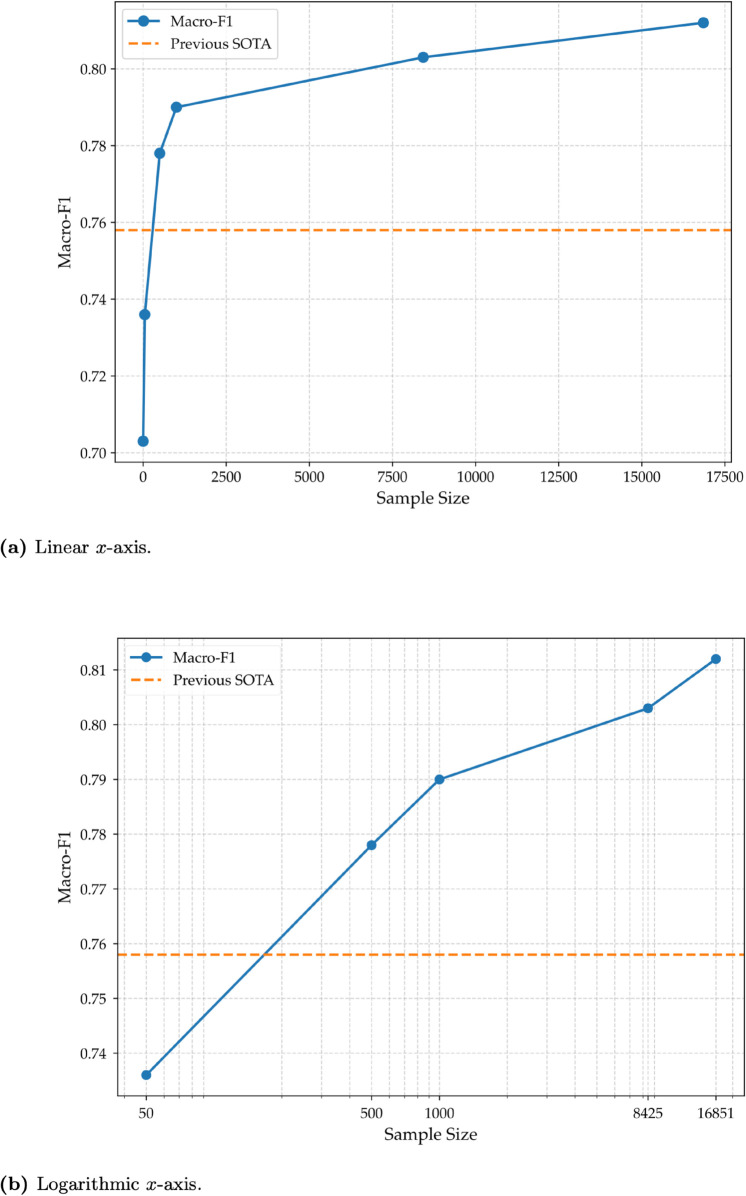
Performance of GPT-4o mini on ArSAS with varying fine-tuning sample sizes. Both figures illustrate the relationship between macro-F1 and fine-tuning sample sizes. (a) The relationship shown with a linear *x*-axis. (b) The same relationship shown with a logarithmic *x*-axis. The *x*-axis shows the fine-tuning sample size, and the *y*-axis shows the macro-F1. The orange dashed line represents the previous SOTA macro-F1 score (0.758).

Fig 7a and Fig 7b illustrate the relationship between macro-F1 and the number of training samples, with different scaling on the x-axis. Both figures demonstrate that macro-F1 improves with increasing sample size, though the rate of improvement diminishes as the dataset grows larger. Both figures highlight that the largest performance gains occur with small sample sizes, particularly up to 500 examples. Beyond 1,000 examples, the incremental improvements in macro-F1 shrink considerably. To provide a comprehensive analysis of the model’s scaling behavior, the results are visualized on plots with both linear and logarithmic x-axes, as they offer complementary perspectives. The logarithmic scale (Fig 7b) is particularly effective for assessing performance across different orders of magnitude; it highlights the significant initial gains from limited data and makes overarching trends, such as diminishing returns, easier to observe. In parallel, the linear scale (Fig 7a) better illustrates the absolute performance differences and the steepness of the learning curve as more data is added. In the logarithmic plot, the value zero is excluded because logarithmic scales are mathematically undefined at zero. One potential issue with this analysis is the different batch sizes used (as mentioned in Sect 3).

**Fig 8:**
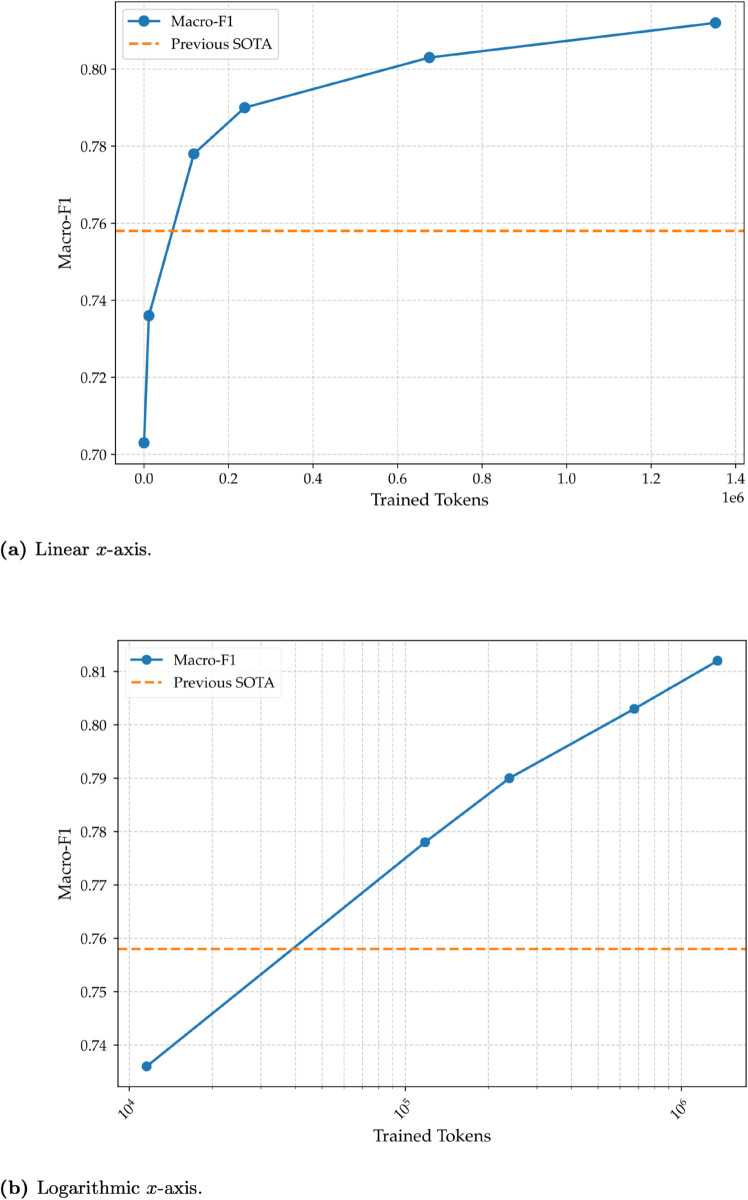
Performance of GPT-4o mini on ArSAS with varying number of trained tokens. Both figures illustrate the relationship between macro-F1 and number of trained tokens. (a) The relationship shown with a linear *x*-axis. (b) The same relationship shown with a logarithmic *x*-axis. The *x*-axis shows the number of trained tokens, and the *y*-axis shows the macro-F1. The orange dashed line represents the previous SOTA macro-F1 score (0.758).

To remove any potential effect this may have, we also constructed two similar figures (to Fig 7a and 7b) with one difference: using the number of trained tokens instead of sample size. This gives a more accurate view since it only accounts for the total number of tokens the model has seen during the fine-tuning stage. It also give a better indication of the price difference between the multiple configurations since cost in fine-tuning LLMs is almost always calculated by the number of tokens. Fig 8a and 8b illustrate the relationship between macro-F1 and the number of trained tokens, with different scaling on the x-axis. We notice a trend similar to Fig 7a and 7b. Fig 8b illustrates clearly the exponential relationship between the number of trained tokens and macro-F1 score; an exponential increase in the number of trained tokens results in a linear increase in the macro-F1 score. As with the previous plot, zero is excluded in the logarithmic plot because logarithmic scales are mathematically undefined at zero. The logarithmic plot in Fig 8b is particularly insightful as it visually suggests that the performance gains follow a predictable scaling law. The near-linear trend on this log-scale plot implies that the relationship between performance and trained tokens is logarithmic. This visual evidence motivates a more formal mathematical modeling of this behavior, which we explore in the Sect 4.4.

#### 4.3.1 Comparative error analysis.

**Fig 9:**
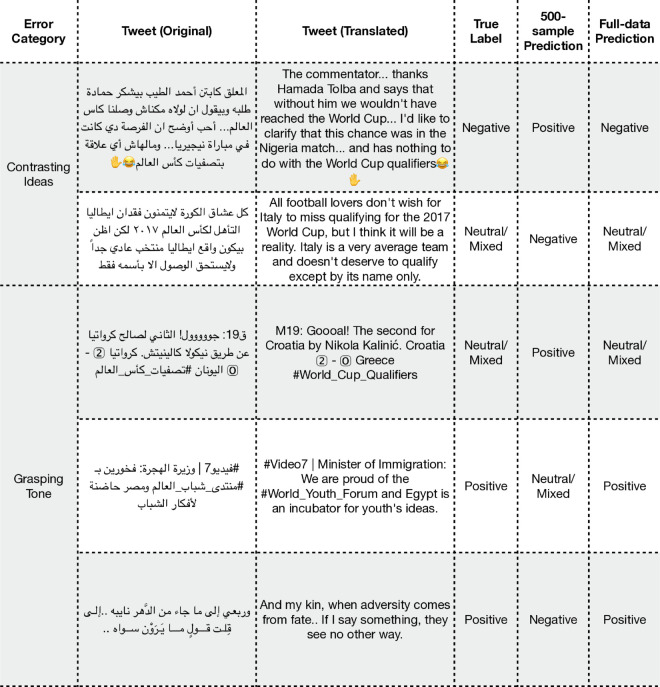
Examples of “fixed errors” for the ArSAS dataset, showing cases where the full-dataset model succeeded but the 500-sample model failed. Note that some tweets include dialectal Arabic, and translations may not fully convey the intended meaning.

While our quantitative results show that model performance improves with more training data, a qualitative analysis offers valuable insights into how its prediction capabilities evolve. To investigate this, we performed a comparative error analysis; we chose to compare two models: the 500-sample model, which represents a highly capable model that already surpasses the previous SOTA, and our strongest model, which was fine-tuned on the entire ArSAS dataset. To see how the model’s capabilities evolved, we investigate the errors made by the 500-sample model that were correctly classified by the full-dataset model. This comparison illustrates the types of subtle errors that more training data can resolve. The discussion below highlights several of these improvements with examples shown in Fig 9.

The observed performance gains appear to correlate with the model’s handling of complex sentences and texts with contrasting sentiments. The 500-sample model, for instance, sometimes produced different classifications than the full-dataset model on some texts. One such case involves a tweet correcting a sports commentator, which the 500-sample model classified as positive. A possible explanation is that the model’s output was influenced by initial words of thanks, without fully integrating the sentence’s main critical point. The full-dataset model, however, produced the correct negative label for this text. A similar pattern can be seen in balanced opinions, which the stronger model correctly labeled as neutral/mixed, whereas the weaker model’s predictions sometimes aligned only with the negative keywords present. Furthermore, the model demonstrated a much better grasp of tone and non-literal language. It appeared less influenced by an exciting keyword like “Goooal!” within a factual, live-sports update, more often identifying the text’s neutral, reportorial function. This improved tonal understanding seems to extend to formal statements and even classical poetry. In these cases, the stronger model could successfully extract the intended positive sentiment where the weaker model failed.

### 4.4 Formalizing performance scaling

We will try in this subsection to formalize the performance scaling we have seen in Fig 8b. We model this relationship using a logarithmic function of the form

F(n)=alog(n)+b, (4)

where *a* is the scaling parameter that determines the rate of improvement with respect to the logarithm of *n*, and *b* is the intercept representing the baseline performance when extrapolated to small values of *n*. Since logarithms are undefined at *n* = 0, we exclude the data point corresponding to *n* = 0 from our analysis. Suppose we have a set of *N* data points $\{(n_{i},F_{i}){\}}_{i=1}^{N}$ with *n*_*i*_>0 and corresponding macro-F1 scores *F*_*i*_. We define the residual sum of squares (RSS) as:

$$E(a,b)=\sum_{i=1}^{N}{\left[F_{i}-\left(a\;\log(n_{i})+b\right)\right]}^{2}.$$ (5)

The optimal parameters (a^*^,b^*^) are obtained by finding the values that minimize this error:

$$(a^{*},b^{*})=\arg\min_{a,b}\;E(a,b).$$ (6)

The data used for the fitting is shown below:

**Table:** 

Trained Tokens (*n*_*i*_)	Macro-F1 (*F*_*i*_)
,562	0.736
,948	0.778
,251	0.790
,333	0.803
,352,170	0.812

Using the non-linear least squares optimization method implemented in curve_fit from scipy.optimize, we find that the minimization of *E*(*a*, *b*) yields

$$a^{*}\approx 0.01604\;\text{and}\;b^{*}\approx 0.58822.$$ (7)

Thus, the fitted model is:

F(n)≈0.01604log(n)+0.58822. (8)

This equation quantifies how the macro-F1 score increases as a function of the number of trained tokens. However, for reasons that will be clear in the rest of the paper, we want the first example *n*_0_ (11,512 trained tokens) to be part of the formalization as a reference point. In our experiments, that macro-F1 score for that point is shown below:

$$F(n_{0})=0.736,$$ (9)

which we will denote by $F_{\text{ref}}$. Two candidate models can be used that include $F_{\text{ref}}$. The first model is given by:

$$F(n)=a\;\log(n)+F_{\text{ref}}+b.$$ (10)

In this formulation both parameters *a* and *b* are free. Although the term $F_{\text{ref}}$ appears, the model does not force the curve to pass through the point (11562,0.736); rather, the best-fit values of *a* and *b* are determined solely by minimizing the error over the data. The second model however explicitly anchors the model at *n*_0_ = 11562 by reparameterizing the logarithmic term:

$$F(n)=a\;\log(\frac{n}{n_{0}})+F_{\text{ref}}+b.$$ (11)

Note that when *n* = 11562, this model yields

$$F(11562)=a\;\log(1)+F_{\text{ref}}+b=F_{\text{ref}}+b.$$ (12)

**Fig 10:**
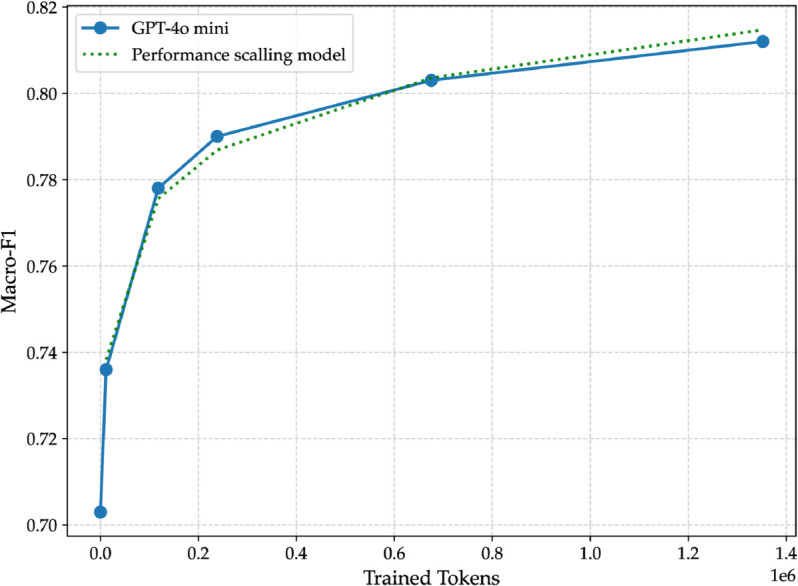
Macro-F1 scores of different fine-tuned versions of GPT-4o mini compared to the performance scaling models (Eq 11). The *x*-axis shows the number of trained tokens, and the *y*-axis shows the macro-F1.

Thus, if we set *b* = 0 (or if the optimization returns a *b* very close to zero), the model is forced to satisfy F(11562)=0.736. Using a non-linear least squares optimization method, we found the optimal parameters for our scaling model (Eq 11) to be $a^{*}\approx 0.01604$ and $b^{*}\approx 0.00231$. As visually confirmed in Fig 10, the resulting curve closely tracks the empirical data points, indicating that the logarithmic model effectively captures the observed performance scaling. This fitted model, which presents the learning curve by plotting the macro-F1 score against the number of training tokens, now serves as a predictive tool. Its primary benefit, as we will demonstrate, is its ability to test whether a different, more powerful LLM follows a similar performance growth trajectory. This question is explored in detail in the following subsection.

### 4.5 Do more powerful LLMs improve performance further?

We wanted to see how a larger and more powerful large language model performs compared to GPT-4o mini. We supplement our experiments with two additional studies using the larger and more powerful GPT-4o model (gpt-4o-2024-08-06).

First, we perform a comparison on the ArSAS dataset between GPT-4o and GPT-4o mini. We fine-tune GPT-4o with 100 and 500 training examples under identical conditions to GPT-4o mini. This includes using identical training and validation sets; and using the same automated hyperparameter selection. Second, we extend our scaling analysis to GPT-4o to investigate whether larger models exhibit the same learning curves. We replicate the progressive scaling approach by fine-tuning on 50 samples. Then we use Eq 11 to predict the values for the rest. After that, we fine-tune on 500 samples, 1,000 samples, and half the dataset (8,425 samples). Due to computational costs, we omit the full 16,851-sample fine-tuning run for GPT-4o. The results we get are then compared to the predicted values to see if GPT-4o follows similar performance scaling to GPT-4o mini.

As shown in Table 7, GPT-4o’s performance exceeds that of GPT-4o mini but not by much; less than one percent for both cases. It’s interesting to note that GPT-4o achieves marginally better results when fine-tuned on 100 samples than GPT-4o mini when fine-tuned on 500 samples. That suggests that when there are less training data available or when the acquisition of even few labeled examples is expensive, a more powerful large language model can learn as well as a weaker one with less data.

**Table 7:** Performance of fine-tuned GPT-4o and GPT-4o mini on ArSAS using 100 and 500 samples. The metric used is macro-F1. ‘Ft.’ in the table refers to fine-tuned.

Model	Ft. (100 samples)	Ft. (500 samples)
GPT-4o mini	0.778	0.781
GPT-4o	**0.783**	**0.790**

Table 8 details the progression of macro-F1 score with varying number of sample sizes for GPT-4o. As we see, the performance keeps improving with the increasing number of samples. GPT-4o consistently outperforms GPT-4o mini across all sample sizes, demonstrating a more efficient adaptation to the ArSAS dataset. For instance, with 50 fine-tuning examples, GPT-4o achieves 0.743, whereas GPT-4o mini reaches 0.736. This trend continues across different sample sizes, with GPT-4o maintaining a superior macro-F1 score. At 500 samples, the performance gap increases, with GPT-4o reaching 0.787, while GPT-4o mini lags slightly behind at 0.778. Similarly, with 1,000 fine-tuning examples, GPT-4o achieves 0.792, surpassing GPT-4o mini’s 0.790. GPT-4o’s advantage remains with 8,425 fine-tuning samples (50% of training data), GPT-4o achieves an F1 score of 0.810, while GPT-4o mini reaches 0.803, maintaining the trend of superior performance for the more powerful model. Notably, even when GPT-4o mini is fine-tuned on the entire dataset (16,851 samples, 100%), it only achieves 0.812, which is only 0.002 higher than GPT-4o’s score at 50% training data. This shows that GPT-4o requires significantly fewer fine-tuning samples to reach comparable or superior performance.

**Table 8:** Performance of GPT-4o on ArSAS with varying fine-tuning sample sizes. The last column (*n*/*N*) shows the percentage of the number of fine-tuning samples over the total number of samples in the training data.

Sample Size	Number of Tokens	Macro-F1	*n*/*N* (%)
0 (no fine-tuning)	0	0.680	0.00%
50	11562	0.743	0.30%
500	117948	0.787	2.97%
1,000	238251	0.792	5.94%
8,425	675333	0.810	50.00%

**Fig 11:**
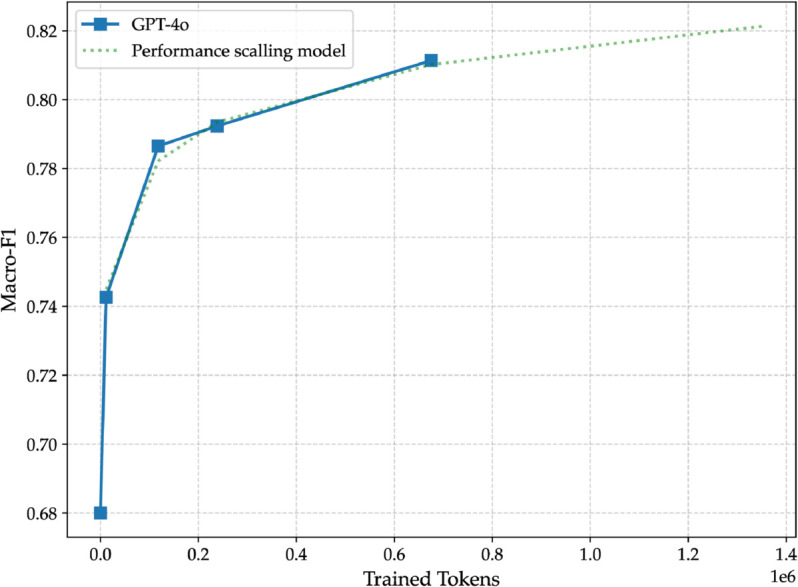
Macro-F1 scores of different fine-tuned versions of GPT-4o compared to the performance scaling models (Eq 11). The *x*-axis shows the number of trained tokens, and the *y*-axis shows the macro-F1.

In Table 9, we present the results of applying Eq 11—derived from GPT-4o mini’s performance growth function—to predict the macro-F1 scores of GPT-4o under different fine-tuning conditions. Remarkably, the predicted results align exceptionally well with the actual fine-tuning performance, exhibiting only minor deviations across all observed sample sizes. A striking aspect of these results is that the growth pattern observed in GPT-4o mini effectively generalizes to GPT-4o, despite being two different models. As seen in Fig 11, the macro-F1 scores of GPT-4o fine-tuned with varying token counts closely track the predictions derived from Eq 11. One of the most surprising insights is the degree of predictive accuracy achieved by the growth function. The absolute difference between predicted and actual macro-F1 scores remains below 0.005 across all sample sizes, with some cases exhibiting perfect agreement between predicted and actual values. Furthermore, these results carry important implications for large-scale LLM experiments. If fine-tuning performance for a larger model can be accurately estimated from a smaller one, researchers and practitioners can use smaller, computationally cheaper models to approximate fine-tuning outcomes before scaling up to larger models. This could significantly reduce the computational and financial cost associated with large-scale fine-tuning experiments.

**Table 9:** Comparison of actual and predicted macro-F1 scores for GPT-4o on ArSAS with different numbers of tokens used for fine-tuning.

Number of Tokens	Actual Macro-F1	Predicted Macro-F1	*Δ*
11562	0.743	0.745	0.002
117948	0.787	0.782	-0.005
238251	0.792	0.793	0.001
675333	0.810	0.810	0.000

### 4.6 Limitations

While our results demonstrate the effectiveness of fine-tuning LLMs with limited data, several limitations should be acknowledged.

First, our experiments were conducted using only GPT-4o mini, GPT-4o, and Gemma, and the results may not generalize to other LLMs. The automated hyperparameter selection, while convenient, may not have produced optimal settings for each task. Finally, budget constraints limited our ability to analyze larger models in a comprehensive manner, perform more scaling experiments, or conduct more extensive hyperparameter optimization.

## 5 Conclusion

This study demonstrates that fine-tuning large language models on small amounts of Arabic training data can yield superior performance compared to existing SOTA models that require full datasets. Our experiments across three different social media tasks show that using just 500 examples (less than 8% of available training data) is sufficient to surpass or match SOTA models. Our analysis of model scaling behavior on the ArSAS dataset reveals that while performance continues to improve with more training data, the most substantial gains occur in the early stages.

These results challenge the conventional wisdom that extensive training data is necessary for achieving SOTA performance in languages like Arabic. The success of our approach suggests that the rich learning capacity of large language models can effectively compensate for limited task-specific training data. This could particularly benefit applications in social media analysis, where language patterns evolve rapidly and the cost of maintaining large annotated datasets can be substantial. This shift can democratize the development process for new tasks, allowing researchers to adapt general-purpose models quickly and at a lower annotation cost.

Future work could explore whether similar results can be achieved across other Arabic NLP tasks and investigate the minimum amount of training data needed for different types of tasks and languages. Additional possible future work is to explore fine-tuned LLM performance scaling more and across different large language models and tasks.

## Supporting information

S1 Appendix Fine-tuning details. (PDF)
