# Supplementary material for: On the effectiveness of limited-data large language model fine-tuning for Arabic
Source: PLoS One. 2025 Oct 8;20(10):e0332419. doi: 10.1371/journal.pone.0332419 (PMC12507264; doi:10.1371/journal.pone.0332419)
Supplement: S1 Appendix — (PDF) [file pone.0332419.s001.pdf]

Appendix: Fine-tuning details

Table 1: Random seeds generated by the OpenAI fine-tuning service for the initial GPT-4o mini experiments.

**Dataset**	**Seed (100 Samples)**	**Seed (500 Samples)**
ArSAS	424401083	539108835
ASND	874499989	1664860769
ArSarcasm	1262552311	2043706116

Table 2: Random seeds generated by the OpenAI fine-tuning service for the initial (100-sample) and multi-run (500-sample) GPT-4o mini experiments.

**Dataset**	**Seed (100 Samples)**	**Seeds (500 Samples)**
ArSAS	424401083	539108835<br>2115890473<br>364956341<br>1216354864<br>966054099
ASND	874499989	1664860769<br>1892932127<br>501869158<br>32175636<br>389311301
ArSarcasm	1262552311	2043706116<br>793167648<br>363975<br>796989214<br>384307960

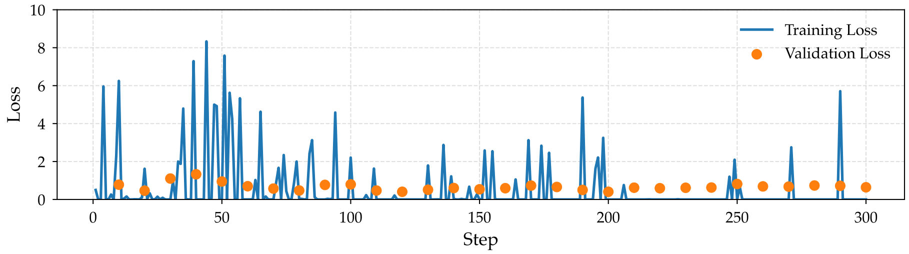

Figure 1: Training and validation loss for the ArSAS model fine-tuned with 100 examples. .

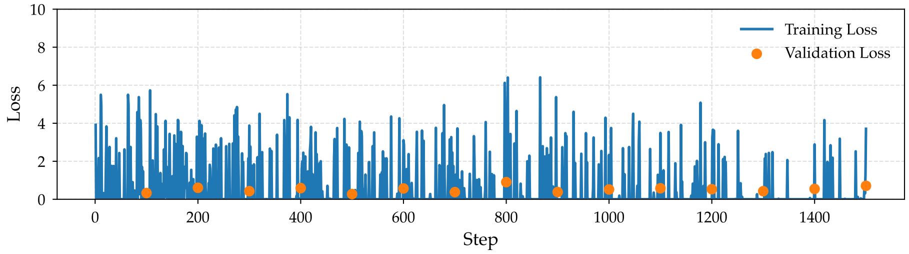

Figure 2: Training and validation loss for the ArSAS model fine-tuned with 500 examples.

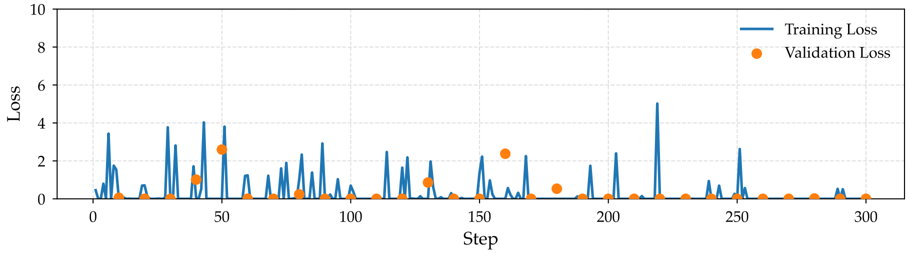

Figure 3: Training and validation loss for the ASND model fine-tuned with 100 examples.

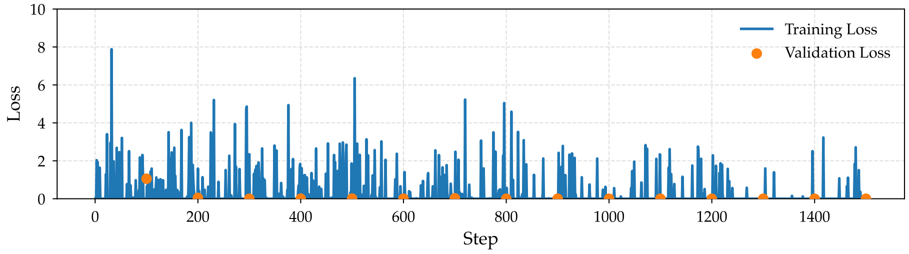

Figure 4: Training and validation loss for the ASND model fine-tuned with 500 examples.

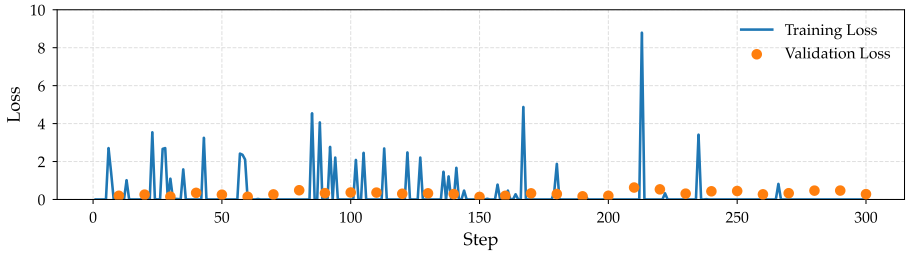

Figure 5: Training and validation loss for the ArSarcasm model fine-tuned with 100 examples.

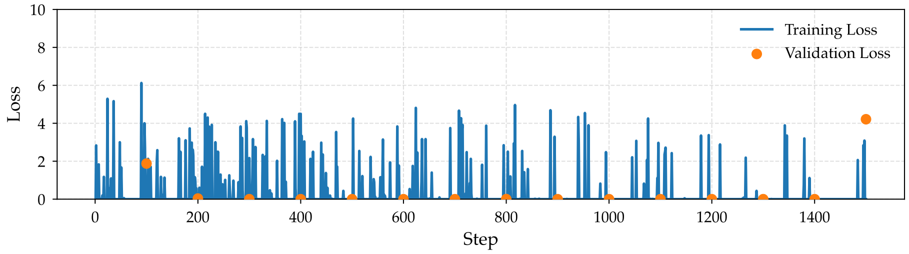

Figure 6: Training and validation loss for the ArSarcasm model fine-tuned with 500 examples.

Table 3: Training and validation loss for the ArSarcasm model fine−tuned with 100 examples from step 1 to 150.

Step	Tr. Loss	Val. Loss	Step	Tr. Loss	Val. Loss	Step	Tr. Loss	Val. Loss
1	0.0005	–	51	0.0001	–	101	0.0006	–
2	0.0000	–	52	0.0003	–	102	2.0841	–
3	0.0000	–	53	0.0002	–	103	0.0003	–
4	0.0000	–	54	0.0003	–	104	0.0002	–
5	0.0000	–	55	0.0009	–	105	2.4586	–
6	2.7084	–	56	0.0019	–	106	0.0002	–
7	1.4214	–	57	2.4181	–	107	0.0006	–
8	0.0000	–	58	2.3563	–	108	0.0002	–
9	0.0000	–	59	2.1054	–	109	0.0004	–
10	0.0000	0.1938	60	0.0011	0.1416	110	0.0011	0.3624
11	0.0000	–	61	0.0545	–	111	0.0009	–
12	0.0000	–	62	0.0006	–	112	0.0007	–
13	1.0162	–	63	0.0013	–	113	2.6889	–
14	0.0006	–	64	0.0266	–	114	0.0008	–
15	0.0016	–	65	0.0003	–	115	0.0006	–
16	0.0003	–	66	0.0002	–	116	0.0004	–
17	0.0001	–	67	0.0034	–	117	0.0009	–
18	0.0004	–	68	0.0026	–	118	0.0003	–
19	0.0179	–	69	0.0061	–	119	0.0003	–
20	0.0025	0.2560	70	0.0000	0.2666	120	0.0003	0.3008
21	0.0035	–	71	0.0001	–	121	0.0002	–
22	0.0004	–	72	0.0000	–	122	2.4796	–
23	3.5418	–	73	0.0000	–	123	0.0002	–
24	0.0062	–	74	0.0000	–	124	0.0001	–
25	0.0010	–	75	0.0000	–	125	0.0000	–
26	0.0004	–	76	0.0000	–	126	0.0001	–
27	2.6669	–	77	0.0000	–	127	2.2088	–
28	2.7088	–	78	0.0000	–	128	0.0000	–
29	0.0010	–	79	0.0000	–	129	0.0001	–
30	1.0963	0.1501	80	0.0000	0.4868	130	0.0000	0.3225
31	0.0335	–	81	0.0000	–	131	0.0001	–
32	0.0338	–	82	0.0000	–	132	0.0001	–
33	0.0242	–	83	0.0000	–	133	0.0000	–
34	0.0088	–	84	0.0000	–	134	0.0000	–
35	1.5865	–	85	4.5417	–	135	0.0000	–
36	0.0019	–	86	0.0000	–	136	1.4626	–
37	0.0018	–	87	0.0000	–	137	0.0000	–
38	0.0013	–	88	4.0625	–	138	1.2172	–
39	0.0064	–	89	0.0000	–	139	0.0002	–
40	0.0061	0.3480	90	0.0000	0.3337	140	0.0000	0.2854
41	0.0011	–	91	0.0001	–	141	1.6690	–
42	0.0001	–	92	2.7710	–	142	0.0000	–
43	3.2502	–	93	0.0002	–	143	0.0000	–
44	0.0001	–	94	2.2089	–	144	0.4689	–
45	0.0001	–	95	0.0006	–	145	0.0000	–
46	0.0001	–	96	0.0024	–	146	0.0000	–
47	0.0000	–	97	0.0043	–	147	0.0000	–
48	0.0001	–	98	0.0049	–	148	0.0000	–
49	0.0001	–	99	0.0015	–	149	0.0069	–
50	0.0002	0.2521	100	0.0012	0.3688	150	0.0000	0.1348

Table 4: Training and validation loss for the ArSarcasm model fine−tuned with 100 examples from step 151 to 300.

Step	Tr. Loss	Val. Loss	Step	Tr. Loss	Val. Loss	Step	Tr. Loss	Val. Loss
151	0.0000	–	201	0.0001	–	251	0.0006	–
152	0.0000	–	202	0.0029	–	252	0.0004	–
153	0.0377	–	203	0.0000	–	253	0.0002	–
154	0.0000	–	204	0.0000	–	254	0.0005	–
155	0.0000	–	205	0.0001	–	255	0.0007	–
156	0.0000	–	206	0.0003	–	256	0.0007	–
157	0.7835	–	207	0.0001	–	257	0.0005	–
158	0.0000	–	208	0.0002	–	258	0.0005	–
159	0.2526	–	209	0.0002	–	259	0.0007	–
160	0.0000	0.1923	210	0.0003	0.6294	260	0.0003	0.2705
161	0.4688	–	211	0.0002	–	261	0.0004	–
162	0.0000	–	212	0.0002	–	262	0.0003	–
163	0.0000	–	213	8.7918	–	263	0.0003	–
164	0.2754	–	214	0.0002	–	264	0.0005	–
165	0.0002	–	215	0.0002	–	265	0.0004	–
166	0.0000	–	216	0.0002	–	266	0.8216	–
167	4.8750	–	217	0.0003	–	267	0.0004	–
168	0.0000	–	218	0.0004	–	268	0.0004	–
169	0.0000	–	219	0.0005	–	269	0.0003	–
170	0.0000	0.3219	220	0.0002	0.5324	270	0.0003	0.3296
171	0.0000	–	221	0.0004	–	271	0.0004	–
172	0.0000	–	222	0.3248	–	272	0.0003	–
173	0.0000	–	223	0.0003	–	273	0.0003	–
174	0.0000	–	224	0.0001	–	274	0.0002	–
175	0.0000	–	225	0.0003	–	275	0.0002	–
176	0.0000	–	226	0.0001	–	276	0.0002	–
177	0.0000	–	227	0.0001	–	277	0.0002	–
178	0.0000	–	228	0.0001	–	278	0.0001	–
179	0.4079	–	229	0.0001	–	279	0.0001	–
180	1.8762	0.2890	230	0.0037	0.3067	280	0.0001	0.4660
181	0.0000	–	231	0.0001	–	281	0.0002	–
182	0.0000	–	232	0.0001	–	282	0.0002	–
183	0.0000	–	233	0.0001	–	283	0.0003	–
184	0.0000	–	234	0.0004	–	284	0.0002	–
185	0.0000	–	235	3.4168	–	285	0.0002	–
186	0.0000	–	236	0.0003	–	286	0.0002	–
187	0.0000	–	237	0.0004	–	287	0.0007	–
188	0.0000	–	238	0.0164	–	288	0.0002	–
189	0.0078	–	239	0.0010	–	289	0.0003	–
190	0.0000	0.1650	240	0.0005	0.4300	290	0.0004	0.4698
191	0.2754	–	241	0.0010	–	291	0.0004	–
192	0.0000	–	242	0.0016	–	292	0.0005	–
193	0.0000	–	243	0.0015	–	293	0.0004	–
194	0.0001	–	244	50.0010	–	294	0.0063	–
195	0.0001	–	245	0.0008	–	295	0.0004	–
196	0.0001	–	246	0.0002	–	296	0.0005	–
197	0.0001	–	247	0.0004	–	297	0.0003	–
198	0.0001	–	248	0.0004	–	298	0.0003	–
199	0.0001	–	249	0.0002	–	299	0.0005	–
200	0.0000	0.1937	250	0.0003	0.4459	300	0.0005	0.2806

Table 5: Training and validation loss for the ArSarcasm model fine−tuned with 500 examples from step 1 to 150.

Step	Tr. Loss	Val. Loss	Step	Tr. Loss	Val. Loss	Step	Tr. Loss	Val. Loss
1	0.0000	–	51	1.6705	–	101	0.0020	–
2	2.8334	–	52	0.0000	–	102	0.0014	–
3	0.0000	–	53	0.0000	–	103	2.1259	–
4	0.0000	–	54	0.0006	–	104	1.9603	–
5	0.0000	–	55	0.0051	–	105	0.0074	–
6	0.0000	–	56	0.0569	–	106	0.0173	–
7	1.8347	–	57	0.0002	–	107	0.0037	–
8	0.0000	–	58	0.0001	–	108	1.7123	–
9	0.0007	–	59	0.0002	–	109	0.0016	–
10	0.0000	–	60	0.0004	–	110	0.0023	–
11	0.0005	–	61	0.0001	–	111	0.0006	–
12	0.0000	–	62	0.0000	–	112	0.0014	–
13	0.0000	–	63	0.0000	–	113	0.0005	–
14	0.1920	–	64	0.0003	–	114	0.0001	–
15	0.0099	–	65	0.0001	–	115	0.0002	–
16	0.0000	–	66	0.0000	–	116	0.0000	–
17	1.1766	–	67	0.0001	–	117	0.0000	–
18	0.0000	–	68	0.0000	–	118	0.0000	–
19	0.0000	–	69	0.0000	–	119	0.0000	–
20	0.0047	–	70	0.0000	–	120	2.0008	–
21	0.0000	–	71	0.0000	–	121	2.5835	–
22	0.0000	–	72	0.0000	–	122	0.0000	–
23	0.0000	–	73	0.0000	–	123	0.0000	–
24	5.2917	–	74	0.0000	–	124	0.0000	–
25	0.0000	–	75	0.0000	–	125	0.0000	–
26	0.0000	–	76	0.0000	–	126	0.0000	–
27	0.5672	–	77	0.0000	–	127	0.0001	–
28	0.0000	–	78	0.0000	–	128	1.1766	–
29	0.0000	–	79	0.0000	–	129	0.0000	–
30	0.5671	–	80	0.0000	–	130	0.1581	–
31	1.0960	–	81	0.0000	–	131	0.0003	–
32	0.0840	–	82	0.0000	–	132	0.2311	–
33	0.0000	–	83	0.0000	–	133	0.0037	–
34	0.7834	–	84	0.0000	–	134	0.0000	–
35	0.5672	–	85	0.0000	–	135	1.1363	–
36	5.1667	–	86	0.0000	–	136	0.0001	–
37	0.0000	–	87	0.0000	–	137	0.0001	–
38	0.0000	–	88	0.0000	–	138	0.0043	–
39	0.0000	–	89	0.0000	–	139	0.0001	–
40	0.0000	–	90	6.1250	–	140	0.0001	–
41	0.0002	–	91	0.0000	–	141	0.0001	–
42	0.0000	–	92	0.0000	–	142	0.0001	–
43	0.0000	–	93	0.0000	–	143	0.0001	–
44	0.0000	–	94	60.0000	–	144	0.0002	–
45	0.0000	–	95	0.0000	–	145	0.0009	–
46	0.0000	–	96	4.0000	–	146	0.0004	–
47	0.0000	–	97	2.7084	–	147	0.0002	–
48	3.0002	–	98	0.0001	–	148	0.0002	–
49	0.0000	–	99	0.0005	–	149	0.0002	–
50	0.0000	–	100	0.0023	1.8762	150	0.0001	–

Table 6: Training and validation loss for the ArSarcasm model fine−tuned with 500 examples from step 151 to 300.

Step	Tr. Loss	Val. Loss	Step	Tr. Loss	Val. Loss	Step	Tr. Loss	Val. Loss
151	0.0001	–	201	0.1057	–	251	0.0012	–
152	0.0000	–	202	0.0852	–	252	0.0014	–
153	0.0000	–	203	0.6034	–	253	1.0168	–
154	0.0000	–	204	0.0313	–	254	0.0131	–
155	0.0002	–	205	0.0144	–	255	0.0038	–
156	0.0002	–	206	0.0020	–	256	0.0018	–
157	0.0001	–	207	0.0014	–	257	0.0002	–
158	0.0005	–	208	1.7107	–	258	0.0003	–
159	0.0013	–	209	0.0004	–	259	0.0001	–
160	0.0003	–	210	0.0007	–	260	0.0000	–
161	0.0002	–	211	0.0007	–	261	1.2578	–
162	0.0016	–	212	0.0005	–	262	0.5007	–
163	3.2085	–	213	0.0001	–	263	0.0000	–
164	0.0017	–	214	4.5012	–	264	0.0026	–
165	0.0015	–	215	0.0022	–	265	0.0000	–
166	2.5014	–	216	0.0027	–	266	0.0000	–
167	0.0025	–	217	0.0041	–	267	0.0001	–
168	0.0019	–	218	0.0015	–	268	0.0000	–
169	0.0019	–	219	4.3040	–	269	0.9767	–
170	0.0020	–	220	0.0020	–	270	0.0000	–
171	0.0021	–	221	0.0009	–	271	0.0000	–
172	0.0011	–	222	3.8239	–	272	0.0000	–
173	0.0006	–	223	0.0006	–	273	0.0000	–
174	0.0004	–	224	0.0005	–	274	0.0000	–
175	0.0005	–	225	0.0005	–	275	0.0000	–
176	3.1047	–	226	0.0001	–	276	0.0000	–
177	0.0005	–	227	3.9169	–	277	0.0000	–
178	0.0004	–	228	0.0003	–	278	0.0000	–
179	0.0003	–	229	0.0002	–	279	0.8984	–
180	0.0003	–	230	0.0001	–	280	0.0000	–
181	0.0005	–	231	0.0000	–	281	0.0000	–
182	0.0005	–	232	0.0000	–	282	0.0000	–
183	0.0009	–	233	0.0001	–	283	3.8334	–
184	3.7301	–	234	3.0001	–	284	0.0000	–
185	0.0006	–	235	0.0000	–	285	0.0000	–
186	0.0006	–	236	2.5002	–	286	3.2292	–
187	0.0008	–	237	0.0000	–	287	0.4690	–
188	2.9804	–	238	0.0000	–	288	0.0000	–
189	0.0028	–	239	2.5002	–	289	0.0000	–
190	2.6062	–	240	0.0001	–	290	0.0000	–
191	2.2948	–	241	0.0001	–	291	0.0163	–
192	0.0039	–	242	0.0001	–	292	0.0001	–
193	0.0125	–	243	1.2171	–	293	0.0001	–
194	1.1601	–	244	71.2986	–	294	4.1133	–
195	0.9391	–	245	0.0018	–	295	3.7754	–
196	0.0278	–	246	0.0018	–	296	0.0042	–
197	0.5685	–	247	0.0069	–	297	0.0078	–
198	0.0687	–	248	0.7835	–	298	0.0000	–
199	0.0395	–	249	0.0038	–	299	0.0000	–
200	0.0124	0.0349	250	0.0378	–	300	2.1672	0.0000

Table 7: Training and validation loss for the ArSarcasm model fine−tuned with 500 examples from step 301 to 450.

Step	Tr. Loss	Val. Loss	Step	Tr. Loss	Val. Loss	Step	Tr. Loss	Val. Loss
301	0.0000	–	351	0.0025	–	401	0.0000	–
302	0.0000	–	352	0.0012	–	402	3.3333	–
303	0.0000	–	353	0.0019	–	403	0.0000	–
304	0.0002	–	354	2.0226	–	404	0.0000	–
305	0.0000	–	355	0.0493	–	405	0.0000	–
306	1.0962	–	356	0.0031	–	406	0.0000	–
307	3.1667	–	357	0.0007	–	407	3.0417	–
308	0.0000	–	358	0.0001	–	408	0.0000	–
309	0.0049	–	359	0.0001	–	409	0.0000	–
310	0.0000	–	360	0.0000	–	410	0.0003	–
311	0.0002	–	361	0.0001	–	411	0.0000	–
312	2.7501	–	362	0.0000	–	412	0.0000	–
313	0.0034	–	363	0.0000	–	413	0.0001	–
314	0.0003	–	364	4.2188	–	414	0.0000	–
315	0.0002	–	365	0.0000	–	415	0.0000	–
316	0.0001	–	366	0.0001	–	416	0.0000	–
317	0.0001	–	367	0.0000	–	417	0.0000	–
318	0.0008	–	368	4.0210	–	418	0.0005	–
319	0.0005	–	369	0.0001	–	419	2.1256	–
320	0.0022	–	370	0.0002	–	420	0.0001	–
321	0.0002	–	371	0.9189	–	421	0.0001	–
322	0.0002	–	372	0.0001	–	422	0.0001	–
323	0.0001	–	373	0.0001	–	423	0.0001	–
324	0.0001	–	374	0.0001	–	424	2.5002	–
325	0.0001	–	375	0.0001	–	425	0.0000	–
326	2.3340	–	376	0.0001	–	426	0.0001	–
327	0.0003	–	377	0.0000	–	427	0.0000	–
328	0.0001	–	378	0.0001	–	428	0.0000	–
329	0.0001	–	379	0.0002	–	429	0.0000	–
330	0.0005	–	380	0.0001	–	430	0.0001	–
331	2.2513	–	381	0.0001	–	431	0.0000	–
332	0.0001	–	382	0.0001	–	432	0.0001	–
333	0.0001	–	383	0.0000	–	433	0.0000	–
334	4.1251	–	384	0.0000	–	434	0.0000	–
335	0.0001	–	385	0.0000	–	435	0.0000	–
336	0.0001	–	386	0.0000	–	436	0.0001	–
337	0.0001	–	387	0.0000	–	437	0.0000	–
338	0.0000	–	388	0.0000	–	438	0.0000	–
339	0.0001	–	389	4.1042	–	439	0.0000	–
340	0.4712	–	390	0.5672	–	440	2.9792	–
341	0.0001	–	391	0.0000	–	441	0.0001	–
342	0.0001	–	392	0.0000	–	442	0.0000	–
343	0.3030	–	393	0.0000	–	443	2.3545	–
344	0.0010	–	394	0.0000	–	444	0.0000	–
345	0.0002	–	395	0.0000	–	445	0.0005	–
346	0.0002	–	396	0.0000	–	446	0.0000	–
347	0.0003	–	397	4.5000	–	447	0.5673	–
348	0.0007	–	398	4.4583	–	448	0.0001	–
349	0.0003	–	399	0.0002	–	449	1.3804	–
350	0.0024	–	400	4.5000	0.0000	450	0.0000	–

Table 8: Training and validation loss for the ArSarcasm model fine−tuned with 500 examples from step 451 to 600.

Step	Tr. Loss	Val. Loss	Step	Tr. Loss	Val. Loss	Step	Tr. Loss	Val. Loss
451	0.0011	–	501	0.0000	–	551	0.0000	–
452	0.6018	–	502	4.2500	–	552	0.0379	–
453	0.0007	–	503	0.0000	–	553	0.0189	–
454	0.0000	–	504	0.0000	–	554	0.0001	–
455	0.0001	–	505	0.0000	–	555	1.1570	–
456	0.0000	–	506	0.0000	–	556	0.1927	–
457	0.1921	–	507	0.0000	–	557	0.0000	–
458	0.0000	–	508	0.0000	–	558	0.0001	–
459	0.0424	–	509	0.0000	–	559	0.9973	–
460	0.0000	–	510	0.0000	–	560	3.1459	–
461	0.0162	–	511	0.0000	–	561	0.0000	–
462	0.0000	–	512	0.0000	–	562	0.2533	–
463	0.0000	–	513	0.0000	–	563	0.1593	–
464	0.0000	–	514	2.5418	–	564	0.1755	–
465	0.0000	–	515	0.0000	–	565	0.0000	–
466	0.0000	–	516	0.0000	–	566	0.0001	–
467	0.0000	–	517	0.0037	–	567	0.0000	–
468	0.0000	–	518	0.0000	–	568	0.0001	–
469	3.5417	–	519	0.0000	–	569	0.0022	–
470	0.0000	–	520	1.2171	–	570	0.0000	–
471	1.7103	–	521	0.0001	–	571	0.0004	–
472	0.0000	–	522	0.0000	–	572	0.0000	–
473	0.0000	–	523	0.0000	–	573	0.0000	–
474	0.0000	–	524	0.0000	–	574	1.9386	–
475	0.0000	–	525	0.0000	–	575	0.0000	–
476	0.0026	–	526	0.0000	–	576	0.0000	–
477	0.0000	–	527	0.0000	–	577	0.0000	–
478	0.0000	–	528	0.0005	–	578	0.0000	–
479	0.0000	–	529	0.0004	–	579	0.0000	–
480	0.0000	–	530	0.0003	–	580	0.0000	–
481	0.0000	–	531	0.0020	–	581	0.0000	–
482	0.0000	–	532	0.0000	–	582	0.0000	–
483	0.4378	–	533	0.0000	–	583	0.0000	–
484	0.0000	–	534	0.0001	–	584	0.0000	–
485	0.0000	–	535	0.0003	–	585	0.0000	–
486	0.0000	–	536	2.2297	–	586	0.0000	–
487	0.0000	–	537	0.4081	–	587	0.0000	–
488	0.0000	–	538	0.0000	–	588	3.8333	–
489	0.0002	–	539	0.0000	–	589	0.0380	–
490	0.0000	–	540	0.0000	–	590	0.1586	–
491	0.0003	–	541	0.0000	–	591	1.7726	–
492	0.0000	–	542	1.0564	–	592	0.0000	–
493	0.0000	–	543	0.8989	–	593	0.0003	–
494	0.0000	–	544	90.0000	–	594	0.0001	–
495	0.0000	–	545	0.1296	–	595	0.0009	–
496	0.0000	–	546	0.0001	–	596	0.0001	–
497	0.0000	–	547	0.0000	–	597	0.0001	–
498	0.0000	–	548	0.0000	–	598	0.0014	–
499	0.0000	–	549	0.0165	–	599	0.0001	–
500	0.0000	0.0000	550	0.0000	–	600	0.0002	0.0001

Table 9: Training and validation loss for the ArSarcasm model fine−tuned with 500 examples from step 601 to 750.

Step	Tr. Loss	Val. Loss	Step	Tr. Loss	Val. Loss	Step	Tr. Loss	Val. Loss
601	0.0001	–	651	0.0360	–	701	0.0000	–
602	0.0004	–	652	0.0002	–	702	0.0000	–
603	0.0001	–	653	0.0003	–	703	0.0000	–
604	0.0002	–	654	0.0032	–	704	0.0000	–
605	0.0001	–	655	1.4028	–	705	0.0000	–
606	0.0000	–	656	0.0002	–	706	0.0000	–
607	0.0000	–	657	0.0057	–	707	0.0000	–
608	0.0001	–	658	0.0001	–	708	4.6667	–
609	0.0000	–	659	0.0001	–	709	0.0000	–
610	0.0000	–	660	0.0001	–	710	0.0000	–
611	0.0000	–	661	0.0010	–	711	0.0000	–
612	0.5346	–	662	0.0000	–	712	4.2708	–
613	0.0009	–	663	0.0000	–	713	0.0000	–
614	0.0000	–	664	0.0000	–	714	3.6357	–
615	0.0001	–	665	0.0000	–	715	3.9167	–
616	0.0000	–	666	0.0000	–	716	0.0000	–
617	0.0000	–	667	0.0000	–	717	0.0007	–
618	1.7526	–	668	0.0000	–	718	0.0010	–
619	0.0000	–	669	0.0000	–	719	0.0000	–
620	0.0000	–	670	0.0939	–	720	0.0001	–
621	0.0005	–	671	0.0000	–	721	0.2998	–
622	0.0000	–	672	0.0000	–	722	0.0000	–
623	0.3008	–	673	0.0000	–	723	2.4796	–
624	4.8125	–	674	0.0000	–	724	0.0000	–
625	0.0000	–	675	0.0000	–	725	0.0001	–
626	0.0000	–	676	0.0000	–	726	0.0001	–
627	2.4588	–	677	0.0000	–	727	0.0003	–
628	0.0000	–	678	0.0000	–	728	0.0001	–
629	0.0000	–	679	0.0000	–	729	0.8409	–
630	0.0000	–	680	0.0000	–	730	0.0016	–
631	0.0000	–	681	0.0000	–	731	2.1257	–
632	0.0000	–	682	0.0000	–	732	0.2999	–
633	0.0001	–	683	0.0000	–	733	0.0001	–
634	0.0000	–	684	0.0000	–	734	0.0016	–
635	0.9010	–	685	0.0000	–	735	0.0040	–
636	3.1668	–	686	0.0000	–	736	0.0003	–
637	0.0000	–	687	0.0000	–	737	0.0001	–
638	0.0000	–	688	0.0000	–	738	0.0001	–
639	0.0000	–	689	0.0000	–	739	0.0001	–
640	0.0001	–	690	0.0000	–	740	0.0001	–
641	0.0000	–	691	3.7500	–	741	0.0000	–
642	0.0001	–	692	0.0000	–	742	0.0000	–
643	3.1668	–	693	0.0000	–	743	0.0234	–
644	0.0002	–	694	0.0000	–	744	0.0000	–
645	0.0296	–	695	0.0000	–	745	0.0000	–
646	0.0001	–	696	0.0007	–	746	0.0000	–
647	0.0204	–	697	0.0000	–	747	0.0000	–
648	0.0002	–	698	0.0002	–	748	0.0031	–
649	0.0001	–	699	0.0000	–	749	0.0000	–
650	0.0116	–	700	0.0000	0.0000	750	0.0000	–

Table 10: Training and validation loss for the ArSarcasm model fine−tuned with 500 examples from step 751 to 900.

Step	Tr. Loss	Val. Loss	Step	Tr. Loss	Val. Loss	Step	Tr. Loss	Val. Loss
751	0.0003	–	801	0.0000	–	851	0.0000	–
752	0.0000	–	802	0.0000	–	852	0.0000	–
753	1.8140	–	803	2.5054	–	853	0.0000	–
754	0.0000	–	804	0.0000	–	854	0.0000	–
755	0.0000	–	805	0.0000	–	855	0.0022	–
756	0.0007	–	806	0.0000	–	856	0.0207	–
757	0.0028	–	807	0.0000	–	857	0.0000	–
758	0.0005	–	808	1.1989	–	858	0.0000	–
759	0.0000	–	809	0.0001	–	859	0.0000	–
760	0.0000	–	810	0.0214	–	860	0.0000	–
761	3.8750	–	811	0.0003	–	861	0.0000	–
762	0.0003	–	812	0.0001	–	862	0.0000	–
763	0.0000	–	813	0.0001	–	863	0.0000	–
764	0.0000	–	814	0.0117	–	864	0.0233	–
765	0.0000	–	815	2.9480	–	865	0.0000	–
766	0.0000	–	816	0.0000	–	866	0.0000	–
767	0.0000	–	817	4.9635	–	867	0.0000	–
768	0.0000	–	818	0.0000	–	868	0.0000	–
769	0.0000	–	819	0.0000	–	869	0.0000	–
770	0.0003	–	820	0.0000	–	870	0.0000	–
771	0.0054	–	821	0.0000	–	871	0.0000	–
772	0.0006	–	822	0.0000	–	872	0.0000	–
773	0.0000	–	823	0.0000	–	873	0.0000	–
774	0.0015	–	824	0.0000	–	874	0.0003	–
775	0.0045	–	825	0.0000	–	875	0.0000	–
776	0.0001	–	826	0.0000	–	876	0.0000	–
777	0.0000	–	827	0.0207	–	877	0.0000	–
778	0.0022	–	828	0.0000	–	878	0.0000	–
779	0.0007	–	829	0.0000	–	879	0.0000	–
780	0.0001	–	830	0.0000	–	880	0.0000	–
781	0.0017	–	831	2.5418	–	881	0.0000	–
782	0.0014	–	832	0.0000	–	882	0.0000	–
783	0.0007	–	833	0.0000	–	883	0.0000	–
784	0.0000	–	834	0.0000	–	884	0.0001	–
785	0.0000	–	835	1.4214	–	885	0.0000	–
786	0.0001	–	836	0.0000	–	886	4.6875	–
787	0.0000	–	837	0.0000	–	887	0.0001	–
788	0.0000	–	838	0.0000	–	888	0.0000	–
789	0.0010	–	839	0.0000	–	889	0.0000	–
790	0.0000	–	840	0.0000	–	890	0.0000	–
791	0.0000	–	841	0.0000	–	891	0.0000	–
792	0.0000	–	842	1.5862	–	892	0.0000	–
793	0.0000	–	843	0.0000	–	893	0.0000	–
794	0.0000	–	844	0.0000	–	894	3.2917	–
795	2.8441	–	845	0.0000	–	895	0.0000	–
796	0.0000	–	846	0.0000	–	896	0.0001	–
797	0.0000	–	847	0.0000	–	897	0.0000	–
798	0.0000	–	848	0.0000	–	898	0.0000	–
799	0.0000	–	849	0.0000	–	899	0.0001	–
800	0.0000	0.0047	850	0.0000	–	900	0.0001	0.0001

Table 11: Training and validation loss for the ArSarcasm model fine−tuned with 500 examples from step 901 to 1050.

Step	Tr. Loss	Val. Loss	Step	Tr. Loss	Val. Loss	Step	Tr. Loss	Val. Loss
901	0.0001	–	951	0.0037	–	1001	0.0000	–
902	0.0001	–	952	0.0000	–	1002	0.0000	–
903	0.0001	–	953	4.5417	–	1003	0.0000	–
904	0.0000	–	954	0.0000	–	1004	0.0000	–
905	0.0000	–	955	0.0000	–	1005	0.0000	–
906	0.0000	–	956	0.0000	–	1006	0.0000	–
907	0.0000	–	957	0.0000	–	1007	0.0000	–
908	0.0000	–	958	0.0000	–	1008	0.0000	–
909	0.0000	–	959	0.0000	–	1009	0.0000	–
910	0.0000	–	960	3.8959	–	1010	0.0000	–
911	0.0000	–	961	0.0234	–	1011	0.0000	–
912	0.0000	–	962	0.0000	–	1012	0.0000	–
913	0.0000	–	963	0.0000	–	1013	0.0000	–
914	0.0000	–	964	0.0000	–	1014	0.0000	–
915	0.0000	–	965	0.0005	–	1015	0.0000	–
916	0.0000	–	966	0.0001	–	1016	0.0000	–
917	0.0000	–	967	0.0002	–	1017	0.0020	–
918	0.0120	–	968	0.0001	–	1018	0.0000	–
919	0.0000	–	969	0.0002	–	1019	0.0000	–
920	0.0000	–	970	0.0005	–	1020	0.0000	–
921	0.0000	–	971	0.0003	–	1021	0.0000	–
922	0.0000	–	972	0.0006	–	1022	0.0002	–
923	0.0040	–	973	0.0015	–	1023	0.0002	–
924	0.0000	–	974	0.0012	–	1024	0.0000	–
925	0.0034	–	975	0.0001	–	1025	0.0003	–
926	0.0000	–	976	0.0007	–	1026	0.0000	–
927	0.0000	–	977	0.0002	–	1027	0.0000	–
928	0.0000	–	978	0.0003	–	1028	0.0000	–
929	0.0000	–	979	0.0001	–	1029	0.0000	–
930	0.0000	–	980	0.0001	–	1030	0.0000	–
931	0.0000	–	981	0.0001	–	1031	0.0000	–
932	0.0000	–	982	0.0000	–	1032	0.0000	–
933	0.0000	–	983	0.8214	–	1033	0.0000	–
934	0.0000	–	984	0.0002	–	1034	0.0000	–
935	0.0000	–	985	0.0000	–	1035	0.0000	–
936	0.0000	–	986	0.0000	–	1036	0.0000	–
937	0.0000	–	987	0.0000	–	1037	0.0058	–
938	0.0000	–	988	0.0000	–	1038	0.0028	–
939	0.0000	–	989	0.0001	–	1039	0.0000	–
940	4.3333	–	990	0.0000	–	1040	0.0000	–
941	3.0625	–	991	0.0000	–	1041	0.0000	–
942	0.0000	–	992	0.0000	–	1042	0.0000	–
943	0.0000	–	993	0.0000	–	1043	0.0000	–
944	0.0001	–	994	2.4794	–	1044	0.0000	–
945	0.0000	–	995	0.0000	–	1045	2.2089	–
946	0.0000	–	996	0.0002	–	1046	0.0004	–
947	0.0088	–	997	0.0000	–	1047	0.0000	–
948	0.0000	–	998	0.0000	–	1048	0.0005	–
949	0.0000	–	999	0.0000	–	1049	0.0002	–
950	0.0000	–	1000	0.0000	0.0000	1050	0.0000	–

Table 12: Training and validation loss for the ArSarcasm model fine−tuned with 500 examples from step 1051 to 1200.

Step	Tr. Loss	Val. Loss	Step	Tr. Loss	Val. Loss	Step	Tr. Loss	Val. Loss
1051	0.0005	–	1101	0.0004	–	1151	0.0005	–
1052	0.0000	–	1102	0.0002	–	1152	0.0013	–
1053	3.0740	–	1103	0.1047	–	1153	0.0006	–
1054	0.0000	–	1104	0.0001	–	1154	0.0002	–
1055	0.0000	–	1105	0.0003	–	1155	0.0002	–
1056	0.0000	–	1106	0.0004	–	1156	0.0001	–
1057	0.0000	–	1107	0.0002	–	1157	0.0003	–
1058	0.0000	–	1108	2.3545	–	1158	0.0002	–
1059	0.0000	–	1109	2.5002	–	1159	0.0001	–
1060	0.0000	–	1110	2.7189	–	1160	0.0003	–
1061	0.0000	–	1111	0.0001	–	1161	0.0002	–
1062	0.0000	–	1112	0.0001	–	1162	0.0002	–
1063	0.0000	–	1113	0.0002	–	1163	0.0001	–
1064	0.0000	–	1114	0.0011	–	1164	0.0001	–
1065	0.0000	–	1115	0.0011	–	1165	0.0000	–
1066	0.0000	–	1116	0.0011	–	1166	0.0001	–
1067	0.0000	–	1117	0.0008	–	1167	0.0001	–
1068	0.8218	–	1118	0.0005	–	1168	0.0001	–
1069	0.0000	–	1119	0.0004	–	1169	0.0001	–
1070	0.0001	–	1120	0.0010	–	1170	0.0001	–
1071	0.0001	–	1121	0.0001	–	1171	0.0000	–
1072	0.0001	–	1122	2.4279	–	1172	0.0000	–
1073	0.0001	–	1123	0.0002	–	1173	0.0001	–
1074	2.0773	–	1124	0.0008	–	1174	0.0001	–
1075	0.0002	–	1125	0.0004	–	1175	0.0004	–
1076	4.2528	–	1126	0.0005	–	1176	0.0001	–
1077	0.0001	–	1127	0.0003	–	1177	0.0002	–
1078	0.0001	–	1128	0.0014	–	1178	0.0001	–
1079	0.0002	–	1129	0.0009	–	1179	3.3543	–
1080	0.0002	–	1130	0.0004	–	1180	0.0020	–
1081	0.0179	–	1131	0.0003	–	1181	0.0002	–
1082	0.0001	–	1132	0.0001	–	1182	0.0005	–
1083	0.0001	–	1133	0.0003	–	1183	0.0006	–
1084	0.0001	–	1134	0.0002	–	1184	0.0007	–
1085	0.0159	–	1135	0.0001	–	1185	0.0003	–
1086	0.0001	–	1136	0.0002	–	1186	0.0004	–
1087	0.0002	–	1137	0.0002	–	1187	0.0008	–
1088	0.0001	–	1138	0.0002	–	1188	0.0026	–
1089	0.0045	–	1139	0.0003	–	1189	0.0021	–
1090	0.0001	–	1140	0.0003	–	1190	0.0033	–
1091	0.0001	–	1141	0.0004	–	1191	0.0011	–
1092	0.0002	–	1142	0.0008	–	1192	0.0031	–
1093	0.0002	–	1143	0.0015	–	1193	0.0006	–
1094	1.1162	–	1144	0.0018	–	1194	3.3761	–
1095	0.0011	–	1145	0.0007	–	1195	0.0049	–
1096	2.9689	–	1146	0.0004	–	1196	0.0034	–
1097	0.0009	–	1147	0.0480	–	1197	0.0024	–
1098	0.0009	–	1148	0.0006	–	1198	0.0018	–
1099	0.0005	–	1149	0.0011	–	1199	0.0011	–
1100	0.0001	0.0002	1150	0.0008	–	1200	0.0007	0.0005

Table 13: Training and validation loss for the ArSarcasm model fine−tuned with 500 examples from step 1201 to 1350.

Step	Tr. Loss	Val. Loss	Step	Tr. Loss	Val. Loss	Step	Tr. Loss	Val. Loss
1201	0.0003	–	1251	0.0000	–	1301	0.0005	–
1202	0.0004	–	1252	0.0002	–	1302	0.0012	–
1203	0.0003	–	1253	0.0000	–	1303	0.0008	–
1204	0.0009	–	1254	0.0001	–	1304	0.0011	–
1205	0.0003	–	1255	0.0009	–	1305	0.0010	–
1206	0.0002	–	1256	0.0013	–	1306	0.0004	–
1207	0.0003	–	1257	0.0001	–	1307	0.0006	–
1208	0.0002	–	1258	0.0026	–	1308	0.0004	–
1209	0.0003	–	1259	0.0007	–	1309	0.0003	–
1210	0.0003	–	1260	0.0359	–	1310	0.0003	–
1211	0.0005	–	1261	0.0356	–	1311	0.0003	–
1212	0.0002	–	1262	0.0830	–	1312	0.0002	–
1213	0.0001	–	1263	0.0325	–	1313	0.0001	–
1214	0.0002	–	1264	0.0119	–	1314	0.0001	–
1215	0.0002	–	1265	0.0003	–	1315	0.0001	–
1216	2.8862	–	1266	2.1883	–	1316	0.0101	–
1217	0.0002	–	1267	0.0001	–	1317	0.0001	–
1218	0.0001	–	1268	0.0015	–	1318	0.0001	–
1219	0.0001	–	1269	0.0007	–	1319	0.0001	–
1220	0.0001	–	1270	0.0004	–	1320	0.0001	–
1221	0.0001	–	1271	0.0002	–	1321	0.0001	–
1222	0.0001	–	1272	0.0004	–	1322	0.0001	–
1223	0.0001	–	1273	0.0010	–	1323	0.0000	–
1224	0.0000	–	1274	0.0006	–	1324	0.0001	–
1225	0.0000	–	1275	0.0002	–	1325	0.0001	–
1226	0.0000	–	1276	0.0004	–	1326	0.0000	–
1227	0.0000	–	1277	0.0003	–	1327	0.0001	–
1228	0.0000	–	1278	0.0005	–	1328	0.0001	–
1229	0.0000	–	1279	0.0005	–	1329	0.0000	–
1230	0.0000	–	1280	0.0008	–	1330	0.0001	–
1231	0.0000	–	1281	0.0003	–	1331	0.0001	–
1232	0.0001	–	1282	0.0010	–	1332	0.0001	–
1233	0.0000	–	1283	0.0006	–	1333	0.0001	–
1234	0.0000	–	1284	0.0002	–	1334	0.0001	–
1235	0.0000	–	1285	0.0005	–	1335	0.0001	–
1236	0.0000	–	1286	0.0004	–	1336	0.0001	–
1237	0.0000	–	1287	0.4381	–	1337	0.0001	–
1238	0.0000	–	1288	0.0004	–	1338	0.0002	–
1239	0.0003	–	1289	0.0004	–	1339	0.0002	–
1240	0.0000	–	1290	0.0021	–	1340	0.0002	–
1241	0.0000	–	1291	0.0005	–	1341	0.0002	–
1242	0.0000	–	1292	0.0005	–	1342	3.8907	–
1243	0.0000	–	1293	0.0006	–	1343	0.0001	–
1244	0.0000	–	1294	0.0005	–	1344	0.0004	–
1245	0.0000	–	1295	0.0009	–	1345	0.0004	–
1246	0.0000	–	1296	0.0006	–	1346	3.3543	–
1247	0.0000	–	1297	0.0004	–	1347	0.0003	–
1248	0.0001	–	1298	0.0043	–	1348	0.0002	–
1249	0.0001	–	1299	0.0006	–	1349	0.0002	–
1250	0.0000	–	1300	0.0006	0.0008	1350	0.0002	–

Table 14: Training and validation loss for the ArSarcasm model fine−tuned with 500 examples from step 1351 to 1500.

Step	Tr. Loss	Val. Loss	Step	Tr. Loss	Val. Loss	Step	Tr. Loss	Val. Loss
1351	0.0002	–	1401	0.0004	–	1451	0.0002	–
1352	0.0002	–	1402	0.0008	–	1452	0.0002	–
1353	0.0003	–	1403	0.0006	–	1453	0.0002	–
1354	0.0003	–	1404	0.0003	–	1454	0.0006	–
1355	0.0002	–	1405	0.0004	–	1455	0.0003	–
1356	0.0002	–	1406	0.0003	–	1456	0.0003	–
1357	0.0002	–	1407	0.0004	–	1457	0.0004	–
1358	0.0002	–	1408	0.0004	–	1458	0.0005	–
1359	0.0003	–	1409	0.0002	–	1459	0.0008	–
1360	0.0002	–	1410	0.0002	–	1460	0.0002	–
1361	0.0005	–	1411	0.0004	–	1461	0.0004	–
1362	0.0002	–	1412	0.0001	–	1462	0.0003	–
1363	0.0003	–	1413	0.0002	–	1463	0.0003	–
1364	0.0002	–	1414	0.0002	–	1464	0.0003	–
1365	0.0002	–	1415	0.0001	–	1465	0.0003	–
1366	0.0002	–	1416	0.0003	–	1466	0.0007	–
1367	0.0006	–	1417	0.0001	–	1467	0.0003	–
1368	0.0002	–	1418	0.0001	–	1468	0.0005	–
1369	0.0002	–	1419	0.0009	–	1469	0.0003	–
1370	0.0002	–	1420	0.0001	–	1470	0.0004	–
1371	0.0002	–	1421	0.0001	–	1471	0.0004	–
1372	0.0002	–	1422	0.0010	–	1472	0.0007	–
1373	0.0003	–	1423	0.0003	–	1473	0.0006	–
1374	0.0003	–	1424	0.0001	–	1474	0.0003	–
1375	0.0011	–	1425	0.0001	–	1475	0.0003	–
1376	0.0004	–	1426	0.0001	–	1476	0.0003	–
1377	0.0004	–	1427	0.0001	–	1477	0.0009	–
1378	0.0004	–	1428	0.0001	–	1478	0.0003	–
1379	0.7657	–	1429	0.0002	–	1479	0.0003	–
1380	3.2012	–	1430	0.0001	–	1480	0.0003	–
1381	0.0008	–	1431	0.0001	–	1481	0.0005	–
1382	0.0008	–	1432	0.0004	–	1482	0.0002	–
1383	0.0006	–	1433	0.0001	–	1483	0.0002	–
1384	0.0006	–	1434	0.0001	–	1484	2.0638	–
1385	0.0013	–	1435	0.0001	–	1485	0.0003	–
1386	0.0013	–	1436	0.0001	–	1486	0.0004	–
1387	0.0011	–	1437	0.0002	–	1487	0.0007	–
1388	0.0012	–	1438	0.0001	–	1488	0.0002	–
1389	0.0007	–	1439	0.0001	–	1489	0.0002	–
1390	1.1167	–	1440	0.0001	–	1490	0.0003	–
1391	0.0011	–	1441	0.0001	–	1491	0.0005	–
1392	0.0016	–	1442	0.0001	–	1492	0.0002	–
1393	0.0008	–	1443	0.0001	–	1493	0.0011	–
1394	0.0006	–	1444	0.0001	–	1494	0.0003	–
1395	0.0009	–	1445	0.0001	–	1495	2.8440	–
1396	0.0008	–	1446	0.0001	–	1496	0.0003	–
1397	0.0008	–	1447	0.0001	–	1497	3.0888	–
1398	0.0007	–	1448	0.0004	–	1498	0.0005	–
1399	0.0008	–	1449	0.0002	–	1499	0.0007	–
1400	0.0008	0.0007	1450	0.0003	–	1500	0.0004	4.2132

Table 15: Training and validation loss for the arSAS model fine−tuned with 100 examples from step 1 to 150.

Step	Tr. Loss	Val. Loss	Step	Tr. Loss	Val. Loss	Step	Tr. Loss	Val. Loss
1	0.5007	–	51	7.5845	–	101	0.0000	–
2	0.0003	–	52	0.0003	–	102	0.0007	–
3	0.0002	–	53	5.6250	–	103	0.0028	–
4	5.9584	–	54	4.2501	–	104	0.0000	–
5	0.0009	–	55	0.0001	–	105	0.0008	–
6	0.0005	–	56	0.0000	–	106	0.2319	–
7	0.2622	–	57	5.3336	–	107	0.0026	–
8	0.0000	–	58	0.0006	–	108	0.0000	–
9	2.3067	–	59	0.0005	–	109	1.6290	–
10	6.2500	0.7802	60	0.0023	0.6988	110	0.0002	0.4699
11	0.0001	–	61	0.0011	–	111	0.0002	–
12	0.0436	–	62	0.0066	–	112	0.0005	–
13	0.1581	–	63	1.0258	–	113	0.0003	–
14	0.0000	–	64	0.0089	–	114	0.0028	–
15	0.0001	–	65	4.6260	–	115	0.0000	–
16	0.0101	–	66	0.0042	–	116	0.0003	–
17	0.0104	–	67	0.1587	–	117	0.2118	–
18	0.0034	–	68	0.0019	–	118	0.0001	–
19	0.1080	–	69	0.0029	–	119	0.0002	–
20	1.6199	0.4637	70	0.0006	0.5736	120	0.0007	0.4051
21	0.0300	–	71	0.8195	–	121	0.0005	–
22	0.3256	–	72	1.6691	–	122	0.0069	–
23	0.0001	–	73	0.0006	–	123	0.0001	–
24	0.0013	–	74	2.3420	–	124	0.0022	–
25	0.1481	–	75	0.4386	–	125	0.0002	–
26	0.0000	–	76	0.0025	–	126	0.0014	–
27	0.0903	–	77	0.0002	–	127	0.0003	–
28	0.0000	–	78	0.8689	–	128	0.0006	–
29	0.0001	–	79	2.0017	–	129	0.0002	–
30	0.0000	1.1027	80	0.0357	0.4730	130	1.7946	0.5112
31	0.9387	–	81	0.0291	–	131	0.0007	–
32	0.0000	–	82	0.0032	–	132	0.0003	–
33	2.0010	–	83	0.0139	–	133	0.0006	–
34	1.8765	–	84	2.4178	–	134	0.0062	–
35	4.7940	–	85	3.1288	–	135	0.0002	–
36	0.0163	–	86	0.1228	–	136	2.8754	–
37	0.0007	–	87	0.0004	–	137	0.0001	–
38	0.0000	–	88	0.0007	–	138	0.0002	–
39	7.2917	–	89	0.0001	–	139	1.2194	–
40	0.0000	1.3276	90	0.0000	0.7682	140	0.0212	0.6025
41	0.0000	–	91	0.0343	–	141	0.0019	–
42	0.0000	–	92	0.0162	–	142	0.0009	–
43	0.0000	–	93	0.0488	–	143	0.0268	–
44	8.3334	–	94	4.5833	–	144	0.0036	–
45	0.0000	–	95	0.0003	–	145	0.0002	–
46	0.0674	–	96	0.0000	–	146	0.6749	–
47	5.0003	–	97	0.0000	–	147	0.0070	–
48	4.9202	–	98	0.0001	–	148	0.0013	–
49	0.0118	–	99	0.0000	–	149	0.2754	–
50	0.0024	0.9465	100	2.2089	0.7951	150	0.0003	0.5362

Table 16: Training and validation loss for the arSAS model fine−tuned with 100 examples from step 151 to 300.

Step	Tr. Loss	Val. Loss	Step	Tr. Loss	Val. Loss	Step	Tr. Loss	Val. Loss
151	0.0001	–	201	0.0000	–	251	0.6726	–
152	2.5839	–	202	0.0001	–	252	0.0000	–
153	0.0001	–	203	0.0044	–	253	0.0000	–
154	0.0002	–	204	0.0000	–	254	0.0000	–
155	2.5421	–	205	0.0000	–	255	0.0000	–
156	0.0001	–	206	0.7610	–	256	0.0000	–
157	0.0002	–	207	0.0000	–	257	0.0000	–
158	0.0001	–	208	0.0001	–	258	0.0000	–
159	0.0002	–	209	0.0000	–	259	0.0000	–
160	0.0001	0.5946	210	0.0000	0.6203	260	0.0000	0.6885
161	0.0008	–	211	0.0000	–	261	0.0000	–
162	0.0001	–	212	0.0000	–	262	0.0001	–
163	0.0001	–	213	0.0017	–	263	0.0000	–
164	1.0562	–	214	0.0000	–	264	0.0000	–
165	0.0011	–	215	0.0000	–	265	0.0000	–
166	0.0002	–	216	0.0058	–	266	0.0000	–
167	0.0002	–	217	0.0000	–	267	0.0000	–
168	0.0003	–	218	0.0000	–	268	0.0000	–
169	3.1262	–	219	0.0003	–	269	0.0000	–
170	0.0015	0.7311	220	0.0000	0.5933	270	0.0002	0.6768
171	0.0008	–	221	0.0014	–	271	2.7501	–
172	0.0007	–	222	0.0000	–	272	0.0001	–
173	0.0016	–	223	0.0000	–	273	0.0001	–
174	2.8345	–	224	0.0001	–	274	0.0001	–
175	0.0013	–	225	0.0000	–	275	0.0001	–
176	0.0012	–	226	0.0004	–	276	0.0001	–
177	2.4594	–	227	0.0127	–	277	0.0001	–
178	0.0009	–	228	0.0000	–	278	0.0001	–
179	0.0009	–	229	0.0000	–	279	0.0001	–
180	0.0009	0.6586	230	0.0000	0.6164	280	0.0001	0.7360
181	0.0009	–	231	0.0000	–	281	0.0001	–
182	0.0018	–	232	0.0000	–	282	0.0001	–
183	0.0005	–	233	0.0000	–	283	0.0001	–
184	0.0011	–	234	0.0000	–	284	0.0001	–
185	0.0003	–	235	0.0000	–	285	0.0002	–
186	0.0015	–	236	0.0001	–	286	0.0001	–
187	0.0011	–	237	0.0000	–	287	0.0001	–
188	0.0004	–	238	0.0003	–	288	0.0000	–
189	0.0008	–	239	0.0000	–	289	0.0002	–
190	5.3753	0.5017	240	0.0000	0.6286	290	5.7084	0.7182
191	0.0003	–	241	0.0000	–	291	0.0001	–
192	0.0004	–	242	0.0000	–	292	0.0001	–
193	0.0007	–	243	0.0000	–	293	0.0000	–
194	0.0003	–	244	0.0000	–	294	0.0000	–
195	1.6294	–	245	0.0000	–	295	0.0000	–
196	2.2138	–	246	0.0000	–	296	0.0000	–
197	0.0407	–	247	1.2015	–	297	0.0001	–
198	3.2501	–	248	0.0021	–	298	0.0042	–
199	0.0000	–	249	2.0945	–	299	0.0000	–
200	0.0000	0.4046	250	0.0000	0.8151	300	0.0001	0.6419

Table 17: Training and validation loss for the arSAS model fine−tuned with 500 examples from step 1 to 150.

Step	Tr. Loss	Val. Loss	Step	Tr. Loss	Val. Loss	Step	Tr. Loss	Val. Loss
1	3.9167	–	51	0.0025	–	101	0.0004	–
2	0.0082	–	52	0.0002	–	102	0.0131	–
3	0.0224	–	53	0.0001	–	103	0.0136	–
4	0.0000	–	54	0.0001	–	104	0.0003	–
5	0.0000	–	55	0.0023	–	105	0.0002	–
6	0.8505	–	56	0.0144	–	106	0.0250	–
7	2.1672	–	57	2.6253	–	107	5.7294	–
8	0.0000	–	58	0.0000	–	108	0.0002	–
9	0.0003	–	59	0.0000	–	109	0.0003	–
10	0.7837	–	60	0.0000	–	110	0.0005	–
11	5.5000	–	61	0.0000	–	111	0.0001	–
12	4.9167	–	62	0.0000	–	112	2.0427	–
13	0.1924	–	63	0.0001	–	113	0.0100	–
14	0.0000	–	64	5.5003	–	114	0.0001	–
15	0.0001	–	65	4.9583	–	115	0.0000	–
16	0.3270	–	66	0.0002	–	116	0.0000	–
17	0.0000	–	67	0.0000	–	117	0.0000	–
18	0.0000	–	68	1.0164	–	118	4.4792	–
19	0.0000	–	69	2.7538	–	119	0.0001	–
20	0.0086	–	70	0.0000	–	120	0.0631	–
21	2.0678	–	71	0.2622	–	121	3.8335	–
22	3.8334	–	72	0.2518	–	122	0.4675	–
23	1.5037	–	73	0.9004	–	123	0.0002	–
24	0.0000	–	74	0.0196	–	124	0.0006	–
25	0.0443	–	75	0.0243	–	125	0.0033	–
26	0.0000	–	76	0.0051	–	126	0.0004	–
27	0.0007	–	77	0.4613	–	127	1.3822	–
28	2.7530	–	78	0.0187	–	128	0.0003	–
29	0.0000	–	79	0.3603	–	129	0.8236	–
30	0.0000	–	80	0.0692	–	130	0.0004	–
31	0.0005	–	81	0.1189	–	131	0.3682	–
32	1.7578	–	82	4.5834	–	132	0.0058	–
33	0.0002	–	83	0.0078	–	133	0.0192	–
34	1.2578	–	84	3.3752	–	134	2.6263	–
35	0.0003	–	85	5.3751	–	135	0.0900	–
36	1.7517	–	86	3.5028	–	136	0.2515	–
37	0.0000	–	87	0.2708	–	137	4.1272	–
38	0.0002	–	88	4.1670	–	138	0.1684	–
39	0.0001	–	89	3.7105	–	139	1.7147	–
40	0.0574	–	90	0.0143	–	140	0.0048	–
41	3.2105	–	91	1.7123	–	141	0.0225	–
42	0.0022	–	92	0.0034	–	142	0.0083	–
43	0.0018	–	93	1.4874	–	143	0.6413	–
44	0.4381	–	94	0.1432	–	144	1.6764	–
45	0.0048	–	95	0.0622	–	145	3.4412	–
46	0.0008	–	96	0.0011	–	146	0.0015	–
47	2.4336	–	97	0.3224	–	147	0.0736	–
48	0.0000	–	98	0.0004	–	148	0.0512	–
49	0.0047	–	99	0.2478	–	149	0.0020	–
50	0.0025	–	100	0.0546	0.3289	150	1.2115	–

Table 18: Training and validation loss for the arSAS model fine−tuned with 500 examples from step 151 to 300.

Step	Tr. Loss	Val. Loss	Step	Tr. Loss	Val. Loss	Step	Tr. Loss	Val. Loss
151	0.0293	–	201	3.0218	–	251	0.0142	–
152	0.0018	–	202	2.0845	–	252	0.0003	–
153	0.0029	–	203	4.1254	–	253	0.0001	–
154	3.3580	–	204	0.0003	–	254	0.0001	–
155	0.0027	–	205	0.0003	–	255	2.7928	–
156	2.9199	–	206	0.0002	–	256	0.0018	–
157	0.0022	–	207	3.8967	–	257	0.0000	–
158	0.0073	–	208	1.7137	–	258	0.0022	–
159	0.0024	–	209	0.0003	–	259	0.0003	–
160	0.0009	–	210	0.0003	–	260	0.0000	–
161	4.1725	–	211	0.0014	–	261	2.9168	–
162	4.1262	–	212	3.2093	–	262	0.1291	–
163	0.0008	–	213	0.0732	–	263	0.1581	–
164	0.0010	–	214	3.4587	–	264	0.0000	–
165	0.0009	–	215	0.6101	–	265	0.0000	–
166	0.0038	–	216	0.0005	–	266	0.0000	–
167	3.5486	–	217	0.0009	–	267	0.0000	–
168	0.0093	–	218	0.0020	–	268	2.9382	–
169	0.0106	–	219	0.0332	–	269	0.0000	–
170	2.8062	–	220	0.0236	–	270	0.0005	–
171	0.0043	–	221	0.8549	–	271	4.4167	–
172	0.0085	–	222	0.3129	–	272	0.0378	–
173	0.0011	–	223	1.8163	–	273	0.0001	–
174	1.7121	–	224	1.5958	–	274	4.7292	–
175	0.5900	–	225	0.0019	–	275	0.0004	–
176	0.7493	–	226	0.0339	–	276	4.8542	–
177	2.4384	–	227	0.0565	–	277	0.0004	–
178	2.3977	–	228	0.1834	–	278	3.3126	–
179	0.0007	–	229	0.2408	–	279	1.5863	–
180	0.0007	–	230	2.6703	–	280	0.0000	–
181	0.0092	–	231	0.8389	–	281	0.0000	–
182	0.0010	–	232	0.0212	–	282	0.0000	–
183	0.0086	–	233	0.0004	–	283	0.0001	–
184	0.8758	–	234	0.0083	–	284	0.0001	–
185	0.0017	–	235	2.8757	–	285	0.0024	–
186	0.0027	–	236	0.0013	–	286	2.4170	–
187	1.5494	–	237	1.2100	–	287	0.0304	–
188	0.0415	–	238	0.0110	–	288	1.9653	–
189	0.0797	–	239	0.0028	–	289	0.0009	–
190	0.0003	–	240	0.0006	–	290	0.0074	–
191	1.0569	–	241	2.7222	–	291	0.0101	–
192	0.0070	–	242	0.0005	–	292	0.0012	–
193	0.7840	–	243	0.0111	–	293	0.0012	–
194	0.0009	–	244	0.0390	–	294	0.0033	–
195	0.3492	–	245	3.2113	–	295	0.0152	–
196	0.0010	–	246	0.0037	–	296	0.0012	–
197	0.0009	–	247	0.0021	–	297	0.0011	–
198	3.2934	–	248	0.0042	–	298	2.1478	–
199	3.0500	–	249	0.0004	–	299	0.0009	–
200	0.0002	0.6043	250	0.0010	–	300	3.8790	0.4267

Table 19: Training and validation loss for the arSAS model fine−tuned with 500 examples from step 301 to 450.

Step	Tr. Loss	Val. Loss	Step	Tr. Loss	Val. Loss	Step	Tr. Loss	Val. Loss
301	0.0016	–	351	0.0001	–	401	0.0176	–
302	2.7743	–	352	0.0001	–	402	1.6147	–
303	0.0032	–	353	0.0001	–	403	1.9918	–
304	1.7756	–	354	3.3959	–	404	0.0006	–
305	0.0043	–	355	0.0003	–	405	2.4529	–
306	0.0059	–	356	0.0001	–	406	0.0001	–
307	0.0043	–	357	0.0001	–	407	0.0026	–
308	0.0078	–	358	0.0001	–	408	0.1225	–
309	1.8396	–	359	0.0000	–	409	2.2547	–
310	0.0033	–	360	0.0000	–	410	0.0001	–
311	0.0025	–	361	0.0056	–	411	0.0001	–
312	0.0028	–	362	0.0001	–	412	0.0009	–
313	0.0075	–	363	0.0001	–	413	1.3920	–
314	0.0056	–	364	0.0001	–	414	1.4671	–
315	0.0160	–	365	0.0007	–	415	0.0017	–
316	2.2155	–	366	0.0000	–	416	0.0009	–
317	0.0105	–	367	0.0000	–	417	0.0030	–
318	0.0003	–	368	4.1668	–	418	2.5638	–
319	0.0001	–	369	2.5003	–	419	2.9585	–
320	4.5002	–	370	0.0001	–	420	3.8126	–
321	0.8220	–	371	0.0000	–	421	1.4483	–
322	0.1168	–	372	1.4533	–	422	0.0011	–
323	0.0010	–	373	0.0000	–	423	1.3268	–
324	0.0143	–	374	5.5313	–	424	0.0015	–
325	2.4802	–	375	0.0001	–	425	0.0004	–
326	0.0011	–	376	0.0000	–	426	0.0001	–
327	0.0010	–	377	0.2156	–	427	0.0002	–
328	0.0005	–	378	4.3125	–	428	0.0020	–
329	0.0084	–	379	0.0001	–	429	3.5210	–
330	0.0004	–	380	0.0000	–	430	0.0001	–
331	0.0005	–	381	0.0001	–	431	2.3760	–
332	0.0003	–	382	0.0000	–	432	1.6702	–
333	0.0109	–	383	0.0000	–	433	0.0007	–
334	0.0001	–	384	0.0001	–	434	0.0006	–
335	0.0001	–	385	0.0001	–	435	0.0000	–
336	0.0002	–	386	0.0004	–	436	0.0001	–
337	0.0002	–	387	0.0001	–	437	1.9046	–
338	0.0035	–	388	0.0002	–	438	0.0000	–
339	0.0021	–	389	0.0002	–	439	2.0016	–
340	2.6677	–	390	0.0006	–	440	0.0107	–
341	0.0002	–	391	0.0007	–	441	0.0000	–
342	0.0002	–	392	0.1813	–	442	0.4217	–
343	2.5420	–	393	0.0001	–	443	0.0000	–
344	0.0010	–	394	4.1776	–	444	0.0000	–
345	0.0008	–	395	0.0007	–	445	0.0000	–
346	0.0004	–	396	0.0006	–	446	0.0000	–
347	0.0014	–	397	0.0001	–	447	0.0000	–
348	0.0002	–	398	0.0333	–	448	0.0001	–
349	0.0003	–	399	0.0055	–	449	0.0003	–
350	0.0002	–	400	0.0129	0.5889	450	0.0001	–

Table 20: Training and validation loss for the arSAS model fine−tuned with 500 examples from step 451 to 600.

Step	Tr. Loss	Val. Loss	Step	Tr. Loss	Val. Loss	Step	Tr. Loss	Val. Loss
451	0.0192	–	501	0.1681	–	551	0.0009	–
452	0.7090	–	502	0.0009	–	552	0.0035	–
453	0.0000	–	503	0.0033	–	553	0.0001	–
454	0.0000	–	504	0.0027	–	554	0.0003	–
455	0.0000	–	505	0.0015	–	555	1.3042	–
456	0.0000	–	506	0.4872	–	556	0.0003	–
457	0.0320	–	507	0.0009	–	557	0.0001	–
458	0.0000	–	508	0.0012	–	558	1.4233	–
459	0.0000	–	509	0.0004	–	559	0.0001	–
460	0.0000	–	510	0.0002	–	560	2.5214	–
461	0.0000	–	511	0.0004	–	561	0.0002	–
462	0.0000	–	512	2.4381	–	562	0.0005	–
463	0.0000	–	513	0.0000	–	563	2.1468	–
464	0.0000	–	514	0.0000	–	564	0.0018	–
465	2.2504	–	515	4.0834	–	565	0.0001	–
466	3.1704	–	516	0.0000	–	566	0.0012	–
467	0.0000	–	517	0.0001	–	567	2.1538	–
468	0.0000	–	518	3.5841	–	568	0.0001	–
469	0.0000	–	519	3.3334	–	569	0.0050	–
470	0.0000	–	520	0.0003	–	570	0.0052	–
471	3.7501	–	521	0.0040	–	571	0.0001	–
472	0.0000	–	522	0.5336	–	572	0.0040	–
473	0.0000	–	523	0.0003	–	573	0.0031	–
474	0.0000	–	524	0.0003	–	574	0.0002	–
475	0.0000	–	525	0.4657	–	575	0.0000	–
476	0.0000	–	526	1.2989	–	576	4.3542	–
477	0.0000	–	527	0.0000	–	577	0.0001	–
478	0.0000	–	528	0.0000	–	578	0.0000	–
479	0.0007	–	529	0.0003	–	579	0.0010	–
480	0.0000	–	530	0.0000	–	580	0.0007	–
481	0.0000	–	531	0.0000	–	581	0.0000	–
482	0.0000	–	532	0.0001	–	582	0.0006	–
483	0.0000	–	533	1.9598	–	583	0.0010	–
484	0.0374	–	534	0.0000	–	584	0.0018	–
485	4.2294	–	535	0.0000	–	585	0.0000	–
486	0.0006	–	536	0.0002	–	586	0.0000	–
487	0.0003	–	537	0.0001	–	587	0.0029	–
488	0.0001	–	538	0.0006	–	588	0.2950	–
489	2.8775	–	539	0.0000	–	589	0.0007	–
490	0.0002	–	540	2.7918	–	590	0.0000	–
491	0.0004	–	541	0.0001	–	591	0.0015	–
492	0.0005	–	542	0.0000	–	592	4.2605	–
493	0.0023	–	543	3.5209	–	593	0.0005	–
494	2.7309	–	544	0.0001	–	594	0.0000	–
495	0.0254	–	545	0.0001	–	595	0.0076	–
496	0.0006	–	546	0.0002	–	596	0.0003	–
497	0.0019	–	547	0.0001	–	597	0.0000	–
498	0.0000	–	548	0.0013	–	598	0.0007	–
499	0.0036	–	549	0.0002	–	599	0.0000	–
500	0.0007	0.2739	550	0.0002	–	600	3.1070	0.5693

Table 21: Training and validation loss for the arSAS model fine−tuned with 500 examples from step 601 to 750.

Step	Tr. Loss	Val. Loss	Step	Tr. Loss	Val. Loss	Step	Tr. Loss	Val. Loss
601	2.8649	–	651	0.0011	–	701	0.2233	–
602	0.0000	–	652	0.2778	–	702	1.3031	–
603	0.0001	–	653	0.0005	–	703	0.0053	–
604	0.0002	–	654	0.0141	–	704	0.4770	–
605	0.0004	–	655	0.0001	–	705	3.7294	–
606	1.5866	–	656	0.0003	–	706	0.0111	–
607	0.0004	–	657	0.0005	–	707	0.0408	–
608	0.0002	–	658	0.0003	–	708	0.7608	–
609	0.0004	–	659	0.0000	–	709	1.0171	–
610	2.3758	–	660	0.0001	–	710	0.0222	–
611	0.0000	–	661	0.0000	–	711	0.0017	–
612	0.0003	–	662	0.0000	–	712	0.0082	–
613	0.0016	–	663	3.7612	–	713	0.0020	–
614	0.0000	–	664	0.0000	–	714	1.4705	–
615	0.0018	–	665	0.0000	–	715	0.0001	–
616	0.8599	–	666	0.0000	–	716	0.0001	–
617	0.0000	–	667	0.0000	–	717	0.0017	–
618	0.0637	–	668	0.0000	–	718	0.5843	–
619	0.0001	–	669	0.0000	–	719	0.1456	–
620	0.0289	–	670	1.7984	–	720	0.0004	–
621	0.0000	–	671	1.7105	–	721	0.0001	–
622	0.0000	–	672	0.0000	–	722	0.0000	–
623	0.0002	–	673	0.0139	–	723	0.0006	–
624	0.0001	–	674	0.0000	–	724	0.0002	–
625	1.5045	–	675	0.0000	–	725	0.0000	–
626	3.5524	–	676	3.4793	–	726	0.0001	–
627	0.0003	–	677	0.0003	–	727	0.0001	–
628	0.0001	–	678	0.0000	–	728	0.0000	–
629	0.0005	–	679	4.9584	–	729	1.3397	–
630	0.0005	–	680	1.1366	–	730	0.0366	–
631	0.0000	–	681	0.0000	–	731	0.0001	–
632	0.0004	–	682	0.0000	–	732	0.0003	–
633	0.0001	–	683	0.0000	–	733	0.0001	–
634	0.0009	–	684	0.0004	–	734	0.0001	–
635	0.0015	–	685	0.0005	–	735	0.0001	–
636	3.6142	–	686	0.0008	–	736	0.0001	–
637	0.0001	–	687	1.7519	–	737	3.3206	–
638	0.0016	–	688	0.0005	–	738	2.1880	–
639	3.0837	–	689	0.0004	–	739	0.0001	–
640	0.0007	–	690	3.1464	–	740	0.0000	–
641	0.0003	–	691	0.0000	–	741	0.0001	–
642	0.0004	–	692	0.0017	–	742	0.0001	–
643	0.0014	–	693	3.2718	–	743	1.0165	–
644	0.0015	–	694	0.0003	–	744	0.0001	–
645	0.0006	–	695	2.4590	–	745	0.0000	–
646	0.0002	–	696	0.0010	–	746	0.0000	–
647	0.0043	–	697	1.7523	–	747	1.0570	–
648	0.0012	–	698	0.0032	–	748	0.0000	–
649	0.0003	–	699	0.0004	–	749	0.0000	–
650	0.0006	–	700	0.0008	0.3764	750	0.6368	–

Table 22: Training and validation loss for the arSAS model fine−tuned with 500 examples from step 751 to 900.

Step	Tr. Loss	Val. Loss	Step	Tr. Loss	Val. Loss	Step	Tr. Loss	Val. Loss
751	0.0001	–	801	0.0000	–	851	0.0001	–
752	0.0000	–	802	4.6667	–	852	0.0000	–
753	0.0012	–	803	6.4063	–	853	0.0000	–
754	0.0000	–	804	0.0000	–	854	0.0000	–
755	0.0002	–	805	0.0000	–	855	0.0000	–
756	0.0001	–	806	0.0000	–	856	0.0000	–
757	0.0001	–	807	0.0000	–	857	0.0000	–
758	0.0000	–	808	3.4376	–	858	0.0000	–
759	0.5007	–	809	0.0000	–	859	0.0004	–
760	4.0625	–	810	0.0000	–	860	0.0000	–
761	0.0001	–	811	0.0000	–	861	0.0000	–
762	0.0000	–	812	0.0000	–	862	0.0000	–
763	0.0000	–	813	0.0000	–	863	0.0023	–
764	0.0003	–	814	0.0000	–	864	0.0000	–
765	0.0000	–	815	0.0000	–	865	0.0000	–
766	0.0000	–	816	2.9377	–	866	6.4167	–
767	0.0000	–	817	0.0000	–	867	0.0000	–
768	2.5004	–	818	0.0000	–	868	0.0000	–
769	0.0000	–	819	0.0000	–	869	0.0000	–
770	0.9701	–	820	4.6458	–	870	0.0000	–
771	0.0000	–	821	0.0000	–	871	2.7917	–
772	1.3275	–	822	0.0000	–	872	0.0000	–
773	0.0001	–	823	0.0000	–	873	2.0010	–
774	0.0000	–	824	0.0000	–	874	0.0000	–
775	0.0000	–	825	0.0000	–	875	2.2504	–
776	1.2632	–	826	0.0000	–	876	0.0000	–
777	0.0000	–	827	0.0000	–	877	0.0000	–
778	0.0000	–	828	0.8213	–	878	0.0000	–
779	0.0000	–	829	0.0000	–	879	2.6878	–
780	0.0000	–	830	0.0000	–	880	0.0100	–
781	0.0000	–	831	0.0000	–	881	2.1913	–
782	0.0001	–	832	0.1580	–	882	0.0000	–
783	0.0001	–	833	0.0000	–	883	0.0000	–
784	0.0000	–	834	0.0000	–	884	2.3337	–
785	0.0000	–	835	0.0000	–	885	0.0000	–
786	0.0000	–	836	0.0000	–	886	0.0000	–
787	0.0005	–	837	0.0000	–	887	3.2500	–
788	0.0000	–	838	0.0000	–	888	0.0001	–
789	0.0000	–	839	0.0000	–	889	0.0000	–
790	0.0000	–	840	0.0000	–	890	0.0000	–
791	0.0000	–	841	2.0008	–	891	0.6726	–
792	0.0000	–	842	0.0000	–	892	0.0001	–
793	0.0000	–	843	0.0000	–	893	0.0001	–
794	0.0000	–	844	0.0599	–	894	0.1290	–
795	0.0000	–	845	2.2938	–	895	0.0000	–
796	0.0000	–	846	0.0001	–	896	0.0158	–
797	6.1250	–	847	0.0306	–	897	5.3750	–
798	0.0000	–	848	0.0001	–	898	0.0000	–
799	0.0000	–	849	0.0000	–	899	0.0000	–
800	0.0000	0.9021	850	0.0001	–	900	0.0000	0.3804

Table 23: Training and validation loss for the arSAS model fine−tuned with 500 examples from step 901 to 1050.

Step	Tr. Loss	Val. Loss	Step	Tr. Loss	Val. Loss	Step	Tr. Loss	Val. Loss
901	0.0000	–	951	0.0000	–	1001	0.0000	–
902	0.0002	–	952	0.0000	–	1002	0.0000	–
903	0.0000	–	953	0.0001	–	1003	0.0001	–
904	0.0001	–	954	0.0001	–	1004	3.7500	–
905	0.0000	–	955	3.4795	–	1005	0.0000	–
906	0.0000	–	956	0.0000	–	1006	0.0004	–
907	0.0000	–	957	1.9593	–	1007	0.0000	–
908	0.0000	–	958	0.0000	–	1008	0.0000	–
909	2.7405	–	959	0.0001	–	1009	0.0000	–
910	0.0000	–	960	0.0000	–	1010	0.0011	–
911	0.0000	–	961	0.0038	–	1011	0.0000	–
912	0.0000	–	962	0.0001	–	1012	0.0000	–
913	0.0002	–	963	0.0001	–	1013	0.0000	–
914	0.0000	–	964	0.0000	–	1014	0.0000	–
915	3.4792	–	965	0.0002	–	1015	0.0004	–
916	0.0005	–	966	0.0001	–	1016	0.0000	–
917	3.3293	–	967	0.0015	–	1017	0.0000	–
918	0.0003	–	968	0.0001	–	1018	0.0000	–
919	0.0000	–	969	0.5844	–	1019	0.0000	–
920	0.0024	–	970	3.2500	–	1020	0.1922	–
921	0.0000	–	971	0.0006	–	1021	0.0000	–
922	0.0020	–	972	0.0001	–	1022	0.0000	–
923	0.0000	–	973	0.0003	–	1023	0.0000	–
924	0.0000	–	974	0.0000	–	1024	0.0000	–
925	0.0007	–	975	0.0001	–	1025	0.0000	–
926	0.0005	–	976	0.0001	–	1026	0.0000	–
927	0.0000	–	977	0.0000	–	1027	2.6877	–
928	0.0000	–	978	0.0000	–	1028	0.0000	–
929	0.0002	–	979	0.0000	–	1029	0.0001	–
930	0.0000	–	980	1.8354	–	1030	1.3250	–
931	4.6042	–	981	0.0000	–	1031	0.0000	–
932	0.0000	–	982	0.0000	–	1032	0.0000	–
933	0.0000	–	983	0.0000	–	1033	0.0000	–
934	1.9089	–	984	0.0000	–	1034	0.0000	–
935	0.0000	–	985	2.5419	–	1035	0.0000	–
936	0.0000	–	986	0.0001	–	1036	0.0000	–
937	0.0001	–	987	0.0000	–	1037	0.0000	–
938	0.0000	–	988	0.0001	–	1038	0.0000	–
939	0.0000	–	989	0.0000	–	1039	2.7501	–
940	0.0019	–	990	0.0001	–	1040	0.0000	–
941	0.0000	–	991	0.0001	–	1041	0.0000	–
942	0.0000	–	992	0.0000	–	1042	0.0001	–
943	0.0000	–	993	4.2917	–	1043	0.0000	–
944	0.0000	–	994	0.0000	–	1044	0.0000	–
945	0.0000	–	995	0.0000	–	1045	0.1162	–
946	2.3962	–	996	0.0000	–	1046	3.3754	–
947	0.0000	–	997	0.0000	–	1047	4.5000	–
948	0.0000	–	998	0.0000	–	1048	0.0000	–
949	0.0000	–	999	0.0000	–	1049	0.0005	–
950	0.0000	–	1000	2.3337	0.5164	1050	0.0000	–

Table 24: Training and validation loss for the arSAS model fine−tuned with 500 examples from step 1051 to 1200.

Step	Tr. Loss	Val. Loss	Step	Tr. Loss	Val. Loss	Step	Tr. Loss	Val. Loss
1051	0.0000	–	1101	0.0002	–	1151	0.0000	–
1052	0.0000	–	1102	0.0000	–	1152	0.0000	–
1053	0.0000	–	1103	0.0000	–	1153	0.0000	–
1054	0.0000	–	1104	0.0000	–	1154	0.0000	–
1055	0.7091	–	1105	0.0004	–	1155	0.0000	–
1056	0.0000	–	1106	1.5227	–	1156	0.0000	–
1057	0.0001	–	1107	0.0004	–	1157	0.0000	–
1058	3.0001	–	1108	0.0008	–	1158	0.0000	–
1059	4.0833	–	1109	0.0000	–	1159	0.0000	–
1060	0.0001	–	1110	0.0000	–	1160	0.1922	–
1061	0.0000	–	1111	0.0001	–	1161	0.0000	–
1062	0.0000	–	1112	0.0013	–	1162	0.0000	–
1063	0.0000	–	1113	0.0001	–	1163	0.8217	–
1064	0.0002	–	1114	0.0000	–	1164	0.0001	–
1065	0.0000	–	1115	0.0000	–	1165	0.0000	–
1066	0.0002	–	1116	3.5417	–	1166	0.0000	–
1067	0.0000	–	1117	0.0269	–	1167	1.7313	–
1068	0.0000	–	1118	0.0002	–	1168	0.0001	–
1069	0.0011	–	1119	0.0000	–	1169	0.0001	–
1070	0.0000	–	1120	0.0001	–	1170	0.0001	–
1071	0.0000	–	1121	0.0003	–	1171	2.0217	–
1072	0.0001	–	1122	0.0002	–	1172	1.0562	–
1073	0.0000	–	1123	0.0000	–	1173	0.0000	–
1074	0.0001	–	1124	0.0007	–	1174	0.0007	–
1075	0.0000	–	1125	0.0003	–	1175	0.0000	–
1076	0.0000	–	1126	0.0002	–	1176	0.0000	–
1077	0.0000	–	1127	0.0004	–	1177	0.0000	–
1078	0.0001	–	1128	0.0004	–	1178	5.0833	–
1079	0.0000	–	1129	1.8974	–	1179	0.0000	–
1080	2.6468	–	1130	0.0000	–	1180	0.0002	–
1081	0.0000	–	1131	0.3794	–	1181	0.0003	–
1082	0.0000	–	1132	0.0109	–	1182	0.0000	–
1083	0.0000	–	1133	0.0005	–	1183	0.0000	–
1084	0.0002	–	1134	0.0002	–	1184	0.7086	–
1085	0.0001	–	1135	0.0014	–	1185	0.0003	–
1086	0.0006	–	1136	0.0000	–	1186	0.0402	–
1087	0.3498	–	1137	0.0002	–	1187	3.0001	–
1088	0.0000	–	1138	0.0003	–	1188	0.0000	–
1089	0.0001	–	1139	0.0000	–	1189	0.0012	–
1090	0.0001	–	1140	3.9168	–	1190	0.0000	–
1091	0.0000	–	1141	0.0000	–	1191	0.0005	–
1092	0.0000	–	1142	0.9377	–	1192	0.0004	–
1093	0.0000	–	1143	0.0000	–	1193	0.0000	–
1094	0.0000	–	1144	0.0000	–	1194	0.0000	–
1095	1.2899	–	1145	0.0000	–	1195	0.0018	–
1096	0.0001	–	1146	0.0000	–	1196	0.0000	–
1097	0.0000	–	1147	0.0000	–	1197	0.0000	–
1098	0.0002	–	1148	0.0000	–	1198	0.0034	–
1099	0.0018	–	1149	0.0000	–	1199	0.0019	–
1100	3.4896	0.5786	1150	0.0000	–	1200	0.0000	0.5307

Table 25: Training and validation loss for the arSAS model fine−tuned with 500 examples from step 1201 to 1350.

Step	Tr. Loss	Val. Loss	Step	Tr. Loss	Val. Loss	Step	Tr. Loss	Val. Loss
1201	3.2500	–	1251	3.6042	–	1301	0.0000	–
1202	3.6609	–	1252	0.5677	–	1302	0.0003	–
1203	0.0001	–	1253	0.0000	–	1303	2.1464	–
1204	3.6251	–	1254	0.0000	–	1304	0.0000	–
1205	0.0000	–	1255	0.0000	–	1305	2.3857	–
1206	0.0000	–	1256	0.8434	–	1306	0.0000	–
1207	0.0156	–	1257	0.0014	–	1307	0.0000	–
1208	0.0060	–	1258	0.0000	–	1308	0.0000	–
1209	0.0000	–	1259	0.1243	–	1309	0.0069	–
1210	0.0034	–	1260	0.0020	–	1310	0.0001	–
1211	0.0000	–	1261	0.0018	–	1311	2.4392	–
1212	0.0000	–	1262	0.0006	–	1312	0.0000	–
1213	2.8959	–	1263	0.0001	–	1313	0.0000	–
1214	0.0000	–	1264	0.0012	–	1314	0.0001	–
1215	0.0000	–	1265	0.0014	–	1315	0.0001	–
1216	0.0009	–	1266	0.0000	–	1316	0.0003	–
1217	0.0002	–	1267	0.0000	–	1317	0.0003	–
1218	0.0005	–	1268	0.0000	–	1318	2.4801	–
1219	0.0000	–	1269	0.0006	–	1319	0.0000	–
1220	0.0000	–	1270	0.0005	–	1320	0.0000	–
1221	0.0001	–	1271	0.0000	–	1321	0.0000	–
1222	2.1166	–	1272	0.0000	–	1322	0.0003	–
1223	0.0000	–	1273	0.0000	–	1323	0.0000	–
1224	1.3396	–	1274	0.0000	–	1324	0.0003	–
1225	2.2297	–	1275	0.0000	–	1325	0.0012	–
1226	0.0001	–	1276	0.0003	–	1326	0.0005	–
1227	1.9623	–	1277	0.0062	–	1327	0.0001	–
1228	0.0000	–	1278	0.0000	–	1328	0.0006	–
1229	0.0000	–	1279	0.0037	–	1329	0.0000	–
1230	0.0007	–	1280	0.0000	–	1330	0.0301	–
1231	0.0001	–	1281	0.0005	–	1331	0.0029	–
1232	0.0000	–	1282	0.0000	–	1332	0.0000	–
1233	0.0000	–	1283	0.0006	–	1333	0.0001	–
1234	0.0003	–	1284	0.0000	–	1334	0.0002	–
1235	0.0000	–	1285	0.0003	–	1335	0.0000	–
1236	0.0002	–	1286	0.0000	–	1336	0.0003	–
1237	0.0000	–	1287	0.0000	–	1337	0.0000	–
1238	0.0000	–	1288	0.2673	–	1338	0.0000	–
1239	0.0000	–	1289	0.0000	–	1339	0.0000	–
1240	0.0000	–	1290	0.0000	–	1340	0.0000	–
1241	0.0009	–	1291	0.0003	–	1341	0.0005	–
1242	0.0007	–	1292	0.0056	–	1342	0.0003	–
1243	0.0000	–	1293	0.0000	–	1343	0.0002	–
1244	0.0000	–	1294	0.0000	–	1344	0.0001	–
1245	0.0034	–	1295	0.0000	–	1345	0.0002	–
1246	0.0186	–	1296	0.0000	–	1346	0.0000	–
1247	0.0000	–	1297	0.0005	–	1347	2.0634	–
1248	0.0000	–	1298	0.0003	–	1348	0.0002	–
1249	0.0000	–	1299	0.0000	–	1349	0.0000	–
1250	0.0000	–	1300	0.0000	0.4251	1350	0.0005	–

Table 26: Training and validation loss for the arSAS model fine−tuned with 500 examples from step 1351 to 1500.

Step	Tr. Loss	Val. Loss	Step	Tr. Loss	Val. Loss	Step	Tr. Loss	Val. Loss
1351	0.0000	–	1401	0.0006	–	1451	0.0004	–
1352	0.0000	–	1402	0.0003	–	1452	0.0006	–
1353	0.0001	–	1403	0.0000	–	1453	0.0003	–
1354	0.0002	–	1404	0.0005	–	1454	0.0004	–
1355	0.0001	–	1405	0.0003	–	1455	0.0013	–
1356	0.0004	–	1406	0.0002	–	1456	0.0008	–
1357	0.0009	–	1407	0.0008	–	1457	0.0004	–
1358	0.0024	–	1408	0.0009	–	1458	0.0000	–
1359	0.0001	–	1409	0.0002	–	1459	0.0252	–
1360	0.0001	–	1410	0.0002	–	1460	0.0003	–
1361	0.0000	–	1411	0.0003	–	1461	0.0000	–
1362	0.0000	–	1412	0.0008	–	1462	0.0004	–
1363	0.0001	–	1413	0.0005	–	1463	0.0004	–
1364	0.0006	–	1414	0.0003	–	1464	0.0000	–
1365	0.0000	–	1415	0.0000	–	1465	0.0004	–
1366	0.0002	–	1416	0.0000	–	1466	0.0000	–
1367	0.0000	–	1417	0.0004	–	1467	0.0004	–
1368	0.0000	–	1418	0.0010	–	1468	0.0003	–
1369	0.0000	–	1419	4.1667	–	1469	0.0003	–
1370	0.0001	–	1420	0.0010	–	1470	0.0002	–
1371	0.0000	–	1421	0.0003	–	1471	0.0000	–
1372	0.0000	–	1422	0.0008	–	1472	0.0006	–
1373	0.0000	–	1423	0.0000	–	1473	0.0000	–
1374	0.0000	–	1424	0.0000	–	1474	0.0004	–
1375	0.0002	–	1425	1.9699	–	1475	0.0000	–
1376	0.0001	–	1426	0.0004	–	1476	0.0007	–
1377	0.0000	–	1427	0.0007	–	1477	0.0004	–
1378	0.0002	–	1428	0.0000	–	1478	0.0007	–
1379	0.0000	–	1429	0.0000	–	1479	0.0014	–
1380	0.0000	–	1430	0.0000	–	1480	2.5215	–
1381	0.0000	–	1431	2.8441	–	1481	0.0007	–
1382	0.0002	–	1432	0.0014	–	1482	0.0007	–
1383	0.0001	–	1433	0.0000	–	1483	0.0004	–
1384	0.0002	–	1434	0.0003	–	1484	0.1268	–
1385	0.0002	–	1435	2.1883	–	1485	0.0002	–
1386	0.0000	–	1436	0.0000	–	1486	0.0006	–
1387	0.0001	–	1437	0.0006	–	1487	0.0007	–
1388	0.0001	–	1438	0.0018	–	1488	0.0005	–
1389	0.0001	–	1439	0.0009	–	1489	0.0006	–
1390	0.0002	–	1440	0.0005	–	1490	0.0494	–
1391	0.0681	–	1441	0.0003	–	1491	0.0012	–
1392	0.0000	–	1442	0.0005	–	1492	0.0005	–
1393	0.0002	–	1443	0.0003	–	1493	0.0009	–
1394	0.0000	–	1444	0.0003	–	1494	0.0005	–
1395	0.0003	–	1445	0.0013	–	1495	0.0003	–
1396	0.0002	–	1446	0.0000	–	1496	0.0000	–
1397	0.0003	–	1447	0.0007	–	1497	0.7834	–
1398	0.0000	–	1448	0.0005	–	1498	0.2996	–
1399	0.0001	–	1449	3.1876	–	1499	1.2799	–
1400	2.8856	0.5453	1450	0.0000	–	1500	3.7086	0.7079

Table 27: Training and validation loss for the ASND model fine−tuned with 100 examples from step 1 to 150.

Step	Tr. Loss	Val. Loss	Step	Tr. Loss	Val. Loss	Step	Tr. Loss	Val. Loss
1	0.4561	–	51	3.8066	–	101	0.3763	–
2	0.0008	–	52	0.0102	–	102	0.0000	–
3	0.0000	–	53	0.0000	–	103	0.0000	–
4	0.7966	–	54	0.0000	–	104	0.0000	–
5	0.0314	–	55	0.0000	–	105	0.0000	–
6	3.4377	–	56	0.0000	–	106	0.0000	–
7	0.0000	–	57	0.0001	–	107	0.0000	–
8	1.7500	–	58	0.0009	–	108	0.0011	–
9	1.5417	–	59	1.2093	–	109	0.0001	–
10	0.0001	0.0544	60	1.2360	0.0065	110	0.0000	0.0001
11	0.0000	–	61	0.0035	–	111	0.0000	–
12	0.0631	–	62	0.0017	–	112	0.0000	–
13	0.0000	–	63	0.0003	–	113	0.0003	–
14	0.0132	–	64	0.0000	–	114	2.4688	–
15	0.0000	–	65	0.0000	–	115	0.0078	–
16	0.0000	–	66	0.0000	–	116	0.0014	–
17	0.0002	–	67	0.0000	–	117	0.0044	–
18	0.0000	–	68	1.2184	–	118	0.0000	–
19	0.6983	–	69	0.0000	–	119	0.0193	–
20	0.7091	0.0000	70	0.0000	0.0001	120	1.6460	0.0000
21	0.1468	–	71	0.0003	–	121	0.0000	–
22	0.0000	–	72	0.0001	–	122	2.1850	–
23	0.0000	–	73	1.6043	–	123	0.0001	–
24	0.0000	–	74	0.0000	–	124	0.0204	–
25	0.0155	–	75	1.8960	–	125	0.0000	–
26	0.0045	–	76	0.0008	–	126	0.0001	–
27	0.0000	–	77	0.0008	–	127	0.1369	–
28	0.0709	–	78	0.0312	–	128	0.0001	–
29	3.7708	–	79	0.0001	–	129	0.0000	–
30	0.0000	0.0000	80	1.0019	0.2351	130	0.0000	0.8597
31	0.0000	–	81	2.3337	–	131	1.9695	–
32	2.8125	–	82	0.0052	–	132	0.6251	–
33	0.0000	–	83	0.0024	–	133	0.0150	–
34	0.0001	–	84	0.0070	–	134	0.0000	–
35	0.0026	–	85	1.3870	–	135	0.0872	–
36	0.0000	–	86	0.0005	–	136	0.0008	–
37	0.0000	–	87	0.0001	–	137	0.0001	–
38	0.0000	–	88	0.0000	–	138	0.0001	–
39	1.7125	–	89	2.9196	–	139	0.3008	–
40	0.0576	1.0126	90	0.0000	0.0000	140	0.0000	0.0000
41	0.0001	–	91	0.0000	–	141	0.0036	–
42	0.5321	–	92	0.2312	–	142	0.0000	–
43	4.0318	–	93	0.0000	–	143	0.0471	–
44	0.0001	–	94	0.0000	–	144	0.0000	–
45	0.0000	–	95	1.0356	–	145	0.0001	–
46	0.0000	–	96	0.0000	–	146	0.0000	–
47	0.0004	–	97	0.0000	–	147	0.0066	–
48	0.0000	–	98	0.0000	–	148	0.0053	–
49	0.0000	–	99	0.0008	–	149	0.0012	–
50	0.0000	2.5963	100	0.6976	0.0000	150	1.3804	0.0000

Table 28: Training and validation loss for the ASND model fine−tuned with 100 examples from step 151 to 300.

Step	Tr. Loss	Val. Loss	Step	Tr. Loss	Val. Loss	Step	Tr. Loss	Val. Loss
151	2.2266	–	201	0.0009	–	251	2.6252	–
152	0.0000	–	202	0.0003	–	252	0.0000	–
153	0.0000	–	203	2.3966	–	253	0.5682	–
154	0.9739	–	204	0.0002	–	254	0.0001	–
155	0.2350	–	205	0.0007	–	255	0.0001	–
156	0.0000	–	206	0.0005	–	256	0.0005	–
157	0.0000	–	207	0.0002	–	257	0.0001	–
158	0.0000	–	208	0.0002	–	258	0.0000	–
159	0.0027	–	209	0.0004	–	259	0.0002	–
160	0.0010	2.3759	210	0.0008	0.0003	260	0.0007	0.0000
161	0.5693	–	211	0.0005	–	261	0.0001	–
162	0.2163	–	212	0.0005	–	262	0.0005	–
163	0.0012	–	213	0.1468	–	263	0.0000	–
164	0.0009	–	214	0.0001	–	264	0.0001	–
165	0.3169	–	215	0.0001	–	265	0.0018	–
166	0.0003	–	216	0.0000	–	266	0.0000	–
167	0.0001	–	217	0.0001	–	267	0.0009	–
168	2.2501	–	218	0.0004	–	268	0.0001	–
169	0.0000	–	219	5.0218	–	269	0.0000	–
170	0.0000	0.0000	220	0.0010	0.0009	270	0.0001	0.0000
171	0.0001	–	221	0.0010	–	271	0.0005	–
172	0.0000	–	222	0.0014	–	272	0.0000	–
173	0.0001	–	223	0.0006	–	273	0.0002	–
174	0.0001	–	224	0.0019	–	274	0.0000	–
175	0.0002	–	225	0.0011	–	275	0.0002	–
176	0.0003	–	226	0.0033	–	276	0.0000	–
177	0.0008	–	227	0.0005	–	277	0.0000	–
178	0.0018	–	228	0.0017	–	278	0.0000	–
179	0.0008	–	229	0.0004	–	279	0.0001	–
180	0.0006	0.5319	230	0.0005	0.0009	280	0.0014	0.0189
181	0.0003	–	231	0.0012	–	281	0.0000	–
182	0.0002	–	232	0.0015	–	282	0.0000	–
183	0.0001	–	233	0.0005	–	283	0.0000	–
184	0.0001	–	234	0.0005	–	284	0.0000	–
185	0.0001	–	235	0.0003	–	285	0.0001	–
186	0.0000	–	236	0.0001	–	286	0.0001	–
187	0.0001	–	237	0.0002	–	287	0.0001	–
188	0.1309	–	238	0.0001	–	288	0.0000	–
189	0.0007	–	239	0.9381	–	289	0.5281	–
190	0.0001	0.0002	240	0.0000	0.0000	290	0.0007	0.0001
191	0.0002	–	241	0.0000	–	291	0.5082	–
192	0.0003	–	242	0.0000	–	292	0.0001	–
193	1.7430	–	243	0.6945	–	293	0.0002	–
194	0.0001	–	244	0.0000	–	294	0.0001	–
195	0.0002	–	245	0.0000	–	295	0.0003	–
196	0.0005	–	246	0.0000	–	296	0.0003	–
197	0.0010	–	247	0.0000	–	297	0.0003	–
198	0.0014	–	248	0.0000	–	298	0.0026	–
199	0.0006	–	249	0.2651	–	299	0.0006	–
200	0.0005	0.0004	250	0.0025	0.0000	300	0.0008	0.0012

Table 29: Training and validation loss for the ASND model fine−tuned with 500 examples from step 1 to 150.

Step	Tr. Loss	Val. Loss	Step	Tr. Loss	Val. Loss	Step	Tr. Loss	Val. Loss
1	0.0336	–	51	0.0000	–	101	0.6850	–
2	0.0003	–	52	3.2089	–	102	0.0165	–
3	2.0327	–	53	0.0000	–	103	0.9274	–
4	0.0205	–	54	0.0000	–	104	0.0023	–
5	1.9303	–	55	0.0005	–	105	0.0391	–
6	0.0031	–	56	0.0000	–	106	0.0001	–
7	0.0264	–	57	0.0000	–	107	0.0004	–
8	0.0088	–	58	0.0001	–	108	1.4397	–
9	1.6357	–	59	0.0034	–	109	0.0011	–
10	0.0001	–	60	0.0000	–	110	1.5938	–
11	0.0001	–	61	0.0128	–	111	0.0000	–
12	0.0001	–	62	0.7725	–	112	0.0004	–
13	0.0001	–	63	0.0012	–	113	0.2001	–
14	0.0012	–	64	0.0025	–	114	0.0001	–
15	0.0002	–	65	0.0007	–	115	0.4900	–
16	0.0001	–	66	2.5098	–	116	0.0001	–
17	0.0000	–	67	0.0026	–	117	0.0009	–
18	0.0000	–	68	0.5507	–	118	0.5247	–
19	0.0000	–	69	0.7034	–	119	1.1252	–
20	0.0000	–	70	0.0003	–	120	0.0000	–
21	1.2923	–	71	0.0020	–	121	0.0056	–
22	0.0000	–	72	0.0004	–	122	0.0003	–
23	3.4063	–	73	0.0324	–	123	0.9892	–
24	0.0000	–	74	0.0637	–	124	0.0001	–
25	0.0000	–	75	0.0003	–	125	0.0002	–
26	0.0023	–	76	0.0006	–	126	0.0006	–
27	0.0000	–	77	0.0237	–	127	1.0318	–
28	0.0000	–	78	0.0001	–	128	0.0003	–
29	0.0088	–	79	0.9787	–	129	0.0000	–
30	2.9601	–	80	0.0009	–	130	0.0000	–
31	0.0000	–	81	0.0003	–	131	0.0000	–
32	7.8854	–	82	0.0028	–	132	0.9813	–
33	0.0000	–	83	0.0015	–	133	0.0001	–
34	0.0961	–	84	0.0371	–	134	0.0625	–
35	0.0002	–	85	0.4531	–	135	0.0001	–
36	1.9693	–	86	0.0004	–	136	0.0026	–
37	0.0027	–	87	2.1772	–	137	0.0017	–
38	0.4473	–	88	0.0001	–	138	0.4745	–
39	1.0212	–	89	0.0001	–	139	0.0001	–
40	0.1309	–	90	0.0081	–	140	0.0403	–
41	0.0183	–	91	0.0024	–	141	0.0073	–
42	2.6879	–	92	2.3110	–	142	0.0158	–
43	0.0000	–	93	1.3765	–	143	3.5105	–
44	0.0025	–	94	0.0627	–	144	0.0001	–
45	0.0000	–	95	0.0012	–	145	2.1263	–
46	2.2292	–	96	0.0004	–	146	0.0001	–
47	2.4585	–	97	0.0002	–	147	0.0002	–
48	0.0000	–	98	0.0009	–	148	0.0003	–
49	0.0066	–	99	0.0024	–	149	2.4451	–
50	0.0000	–	100	0.0123	1.0538	150	0.0004	–

Table 30: Training and validation loss for the ASND model fine−tuned with 500 examples from step 151 to 300.

Step	Tr. Loss	Val. Loss	Step	Tr. Loss	Val. Loss	Step	Tr. Loss	Val. Loss
151	0.0001	–	201	0.0002	–	251	0.0001	–
152	1.5540	–	202	0.4255	–	252	0.0000	–
153	0.0118	–	203	0.0080	–	253	0.0005	–
154	2.6948	–	204	0.0009	–	254	1.5158	–
155	0.1907	–	205	0.0007	–	255	0.0003	–
156	1.2082	–	206	0.0003	–	256	0.0001	–
157	0.0858	–	207	0.0000	–	257	0.0001	–
158	0.0685	–	208	0.0000	–	258	0.0000	–
159	0.0496	–	209	1.1365	–	259	0.0000	–
160	0.4605	–	210	0.1829	–	260	0.0000	–
161	0.0005	–	211	0.0001	–	261	2.2687	–
162	0.3585	–	212	0.0209	–	262	0.0002	–
163	0.0012	–	213	0.4776	–	263	0.0002	–
164	0.0230	–	214	0.0000	–	264	0.1519	–
165	0.2152	–	215	0.0000	–	265	0.1760	–
166	0.0000	–	216	0.0000	–	266	0.0098	–
167	0.0001	–	217	0.0000	–	267	0.0117	–
168	3.6266	–	218	0.0000	–	268	0.0015	–
169	0.0001	–	219	0.0000	–	269	0.0012	–
170	0.0001	–	220	0.6367	–	270	0.0002	–
171	0.0005	–	221	0.0000	–	271	0.0001	–
172	0.0056	–	222	0.0000	–	272	3.9382	–
173	0.0012	–	223	0.0000	–	273	0.0000	–
174	0.0028	–	224	0.0000	–	274	1.6927	–
175	0.0006	–	225	3.4995	–	275	0.0000	–
176	0.0002	–	226	3.3873	–	276	0.0000	–
177	0.0003	–	227	0.9589	–	277	0.0001	–
178	0.0001	–	228	0.0000	–	278	0.0011	–
179	0.1164	–	229	0.0000	–	279	0.0004	–
180	0.0000	–	230	0.0000	–	280	0.1854	–
181	0.0101	–	231	5.2083	–	281	1.5668	–
182	1.1082	–	232	0.0000	–	282	0.2846	–
183	3.2526	–	233	0.0001	–	283	0.0279	–
184	1.4596	–	234	0.0000	–	284	0.0209	–
185	0.0406	–	235	0.0000	–	285	0.0003	–
186	0.0010	–	236	0.0001	–	286	0.0001	–
187	4.0029	–	237	0.0003	–	287	0.0014	–
188	0.0003	–	238	0.0000	–	288	0.0003	–
189	0.0002	–	239	0.0000	–	289	0.0001	–
190	1.7826	–	240	0.8932	–	290	0.0011	–
191	1.0075	–	241	0.0000	–	291	0.0002	–
192	0.4769	–	242	0.0004	–	292	0.0003	–
193	0.1949	–	243	0.0003	–	293	0.0001	–
194	0.0414	–	244	0.0001	–	294	4.7503	–
195	0.0078	–	245	0.0005	–	295	4.8607	–
196	0.0352	–	246	0.0007	–	296	0.0022	–
197	0.0010	–	247	0.0000	–	297	0.0103	–
198	0.0002	–	248	0.0002	–	298	0.0029	–
199	0.0001	–	249	0.0002	–	299	0.0003	–
200	1.5920	0.0435	250	0.0001	–	300	2.3483	0.0003

Table 31: Training and validation loss for the ASND model fine−tuned with 500 examples from step 301 to 450.

Step	Tr. Loss	Val. Loss	Step	Tr. Loss	Val. Loss	Step	Tr. Loss	Val. Loss
301	0.0001	–	351	1.4312	–	401	0.7494	–
302	0.0001	–	352	1.4483	–	402	1.6101	–
303	0.0005	–	353	2.5445	–	403	0.0378	–
304	0.1176	–	354	0.0030	–	404	1.2329	–
305	0.0009	–	355	0.0004	–	405	0.1496	–
306	0.0011	–	356	0.0785	–	406	0.0670	–
307	0.1822	–	357	0.0007	–	407	0.0174	–
308	0.0004	–	358	0.2658	–	408	1.2505	–
309	1.3047	–	359	0.2087	–	409	0.0009	–
310	0.0001	–	360	0.0109	–	410	0.0063	–
311	1.3918	–	361	0.1468	–	411	0.0007	–
312	1.5483	–	362	0.8333	–	412	1.1267	–
313	0.0121	–	363	0.1351	–	413	0.1931	–
314	1.7332	–	364	0.0387	–	414	0.0009	–
315	0.0000	–	365	0.0083	–	415	0.2399	–
316	0.0344	–	366	0.1046	–	416	0.0004	–
317	0.0000	–	367	0.0001	–	417	0.0000	–
318	1.9175	–	368	0.0147	–	418	0.0002	–
319	1.4083	–	369	0.0001	–	419	0.0001	–
320	0.0011	–	370	0.0011	–	420	0.0002	–
321	0.0054	–	371	0.0000	–	421	1.3562	–
322	0.0001	–	372	0.0002	–	422	0.0001	–
323	0.5990	–	373	0.0001	–	423	0.0001	–
324	0.0001	–	374	0.4777	–	424	0.9656	–
325	2.6537	–	375	0.0001	–	425	0.0000	–
326	0.0003	–	376	4.9375	–	426	0.0008	–
327	1.0815	–	377	0.3195	–	427	0.0000	–
328	0.0001	–	378	0.0000	–	428	0.0002	–
329	0.4587	–	379	0.0000	–	429	0.0622	–
330	0.9896	–	380	0.0000	–	430	2.6515	–
331	0.0110	–	381	0.0000	–	431	0.0022	–
332	0.0015	–	382	1.5696	–	432	0.0072	–
333	0.0638	–	383	1.3022	–	433	0.0009	–
334	0.0006	–	384	0.0001	–	434	0.0174	–
335	0.0002	–	385	2.0947	–	435	0.0542	–
336	0.0005	–	386	0.0001	–	436	0.6380	–
337	0.0001	–	387	0.0001	–	437	0.0035	–
338	0.0001	–	388	0.0002	–	438	0.0019	–
339	0.0002	–	389	0.0003	–	439	0.0008	–
340	0.0004	–	390	0.0004	–	440	0.5240	–
341	0.0032	–	391	0.0006	–	441	0.0151	–
342	0.0035	–	392	0.0002	–	442	0.0211	–
343	0.0017	–	393	0.0005	–	443	0.0003	–
344	0.0007	–	394	0.0001	–	444	0.0001	–
345	0.0008	–	395	0.4267	–	445	0.0000	–
346	0.0008	–	396	0.6963	–	446	0.0000	–
347	0.0006	–	397	0.0007	–	447	0.0000	–
348	0.0005	–	398	1.5738	–	448	0.0000	–
349	2.8060	–	399	1.8364	–	449	1.3243	–
350	0.0001	–	400	0.3692	0.0024	450	1.0194	–

Table 32: Training and validation loss for the ASND model fine−tuned with 500 examples from step 451 to 600.

Step	Tr. Loss	Val. Loss	Step	Tr. Loss	Val. Loss	Step	Tr. Loss	Val. Loss
451	0.0000	–	501	0.0054	–	551	0.0000	–
452	0.0000	–	502	0.0000	–	552	0.0000	–
453	2.9194	–	503	0.0001	–	553	0.0001	–
454	0.0005	–	504	0.0018	–	554	0.6045	–
455	0.0324	–	505	6.3542	–	555	0.0001	–
456	0.0252	–	506	0.0000	–	556	0.0000	–
457	0.0000	–	507	0.0000	–	557	3.0236	–
458	0.0000	–	508	0.0000	–	558	0.0886	–
459	0.0000	–	509	0.0000	–	559	0.0009	–
460	0.0586	–	510	2.9063	–	560	0.0001	–
461	0.0000	–	511	0.0000	–	561	0.0014	–
462	0.0001	–	512	0.0000	–	562	0.0001	–
463	0.0038	–	513	0.0001	–	563	0.0001	–
464	0.0424	–	514	0.0002	–	564	0.0001	–
465	2.3085	–	515	2.3153	–	565	2.0550	–
466	0.0092	–	516	0.0005	–	566	0.0003	–
467	1.9381	–	517	0.0005	–	567	0.0001	–
468	0.0076	–	518	0.0004	–	568	0.0002	–
469	0.0027	–	519	0.0004	–	569	0.0001	–
470	0.0005	–	520	0.2171	–	570	0.0004	–
471	0.0000	–	521	0.0037	–	571	0.0004	–
472	1.4693	–	522	0.0000	–	572	0.0028	–
473	0.0001	–	523	0.6830	–	573	0.0000	–
474	0.0000	–	524	0.0489	–	574	0.0033	–
475	0.0001	–	525	0.0688	–	575	0.0012	–
476	2.9063	–	526	0.0122	–	576	0.0000	–
477	1.7501	–	527	0.0000	–	577	0.0010	–
478	0.0002	–	528	3.1339	–	578	0.0001	–
479	0.0000	–	529	0.0003	–	579	0.0001	–
480	0.0001	–	530	0.0004	–	580	0.0001	–
481	0.0004	–	531	2.2920	–	581	0.0016	–
482	2.9688	–	532	0.0000	–	582	0.0001	–
483	1.6680	–	533	0.0000	–	583	0.0001	–
484	0.0063	–	534	0.0000	–	584	1.0226	–
485	0.0000	–	535	0.0000	–	585	0.0010	–
486	0.0974	–	536	0.0000	–	586	0.0005	–
487	0.0127	–	537	0.0000	–	587	2.3752	–
488	2.3750	–	538	0.2105	–	588	0.0014	–
489	2.8544	–	539	0.0000	–	589	0.0000	–
490	0.0005	–	540	1.2313	–	590	0.0020	–
491	0.0002	–	541	0.0000	–	591	0.0001	–
492	0.0002	–	542	0.0000	–	592	0.0000	–
493	0.0001	–	543	0.0653	–	593	0.0003	–
494	0.0001	–	544	2.5662	–	594	0.6596	–
495	0.0000	–	545	0.0000	–	595	0.0009	–
496	0.0000	–	546	0.1073	–	596	0.0016	–
497	0.3393	–	547	0.0000	–	597	0.0609	–
498	0.0000	–	548	0.0000	–	598	0.0000	–
499	1.8609	–	549	0.0002	–	599	0.0000	–
500	0.0000	0.0075	550	0.0000	–	600	0.0003	0.0002

Table 33: Training and validation loss for the ASND model fine−tuned with 500 examples from step 601 to 750.

Step	Tr. Loss	Val. Loss	Step	Tr. Loss	Val. Loss	Step	Tr. Loss	Val. Loss
601	0.0000	–	651	0.0002	–	701	2.4788	–
602	1.4072	–	652	0.0010	–	702	0.0467	–
603	0.3683	–	653	0.0002	–	703	0.0001	–
604	0.0001	–	654	0.0001	–	704	0.0089	–
605	0.0239	–	655	0.0674	–	705	0.0125	–
606	0.0015	–	656	0.0001	–	706	0.0025	–
607	0.0002	–	657	0.6576	–	707	2.0590	–
608	0.0000	–	658	0.0089	–	708	0.0006	–
609	0.0000	–	659	0.0008	–	709	0.3519	–
610	0.0001	–	660	0.0006	–	710	0.0001	–
611	0.4005	–	661	0.0001	–	711	0.0011	–
612	0.0002	–	662	2.5523	–	712	0.0589	–
613	0.3470	–	663	0.0000	–	713	0.0000	–
614	0.0016	–	664	0.0001	–	714	0.2584	–
615	0.0221	–	665	0.0001	–	715	0.0085	–
616	0.0138	–	666	0.0001	–	716	0.0028	–
617	0.0014	–	667	0.0001	–	717	0.0013	–
618	0.0004	–	668	0.0002	–	718	0.0000	–
619	0.0001	–	669	2.3042	–	719	0.0000	–
620	0.0002	–	670	0.0038	–	720	5.2268	–
621	0.0001	–	671	1.1087	–	721	0.0000	–
622	0.0019	–	672	1.1527	–	722	0.0004	–
623	0.0001	–	673	0.6317	–	723	0.0000	–
624	0.0002	–	674	0.0002	–	724	0.0000	–
625	0.0265	–	675	0.2370	–	725	0.0003	–
626	0.0016	–	676	1.6581	–	726	0.0005	–
627	0.0023	–	677	0.0621	–	727	0.0002	–
628	0.0003	–	678	0.0352	–	728	0.7428	–
629	0.0006	–	679	0.0113	–	729	0.0042	–
630	0.0000	–	680	1.2553	–	730	0.0678	–
631	0.0000	–	681	0.0001	–	731	0.0036	–
632	0.0000	–	682	0.6687	–	732	0.0112	–
633	0.0000	–	683	0.0004	–	733	0.0553	–
634	0.7079	–	684	1.7745	–	734	0.0218	–
635	0.0000	–	685	0.3522	–	735	0.0027	–
636	0.0005	–	686	0.0875	–	736	0.0005	–
637	0.0001	–	687	0.0000	–	737	0.0009	–
638	0.9394	–	688	0.0006	–	738	0.0007	–
639	0.0002	–	689	0.0114	–	739	0.0002	–
640	0.0062	–	690	0.0001	–	740	0.0003	–
641	0.0230	–	691	0.0000	–	741	0.0004	–
642	0.0020	–	692	0.7928	–	742	0.0001	–
643	0.0007	–	693	0.2242	–	743	0.0041	–
644	0.0010	–	694	0.0001	–	744	0.0015	–
645	0.0009	–	695	0.0001	–	745	0.0002	–
646	0.0004	–	696	0.0002	–	746	0.0001	–
647	1.0001	–	697	0.0004	–	747	0.0002	–
648	1.3512	–	698	0.0000	–	748	0.0000	–
649	0.0001	–	699	0.0032	–	749	0.0000	–
650	0.0001	–	700	0.0005	0.0216	750	0.0001	–

Table 34: Training and validation loss for the ASND model fine−tuned with 500 examples from step 751 to 900.

Step	Tr. Loss	Val. Loss	Step	Tr. Loss	Val. Loss	Step	Tr. Loss	Val. Loss
751	3.0682	–	801	0.5711	–	851	0.0242	–
752	0.4603	–	802	0.0002	–	852	0.0001	–
753	0.0302	–	803	0.0003	–	853	0.0000	–
754	0.0000	–	804	0.1243	–	854	1.2626	–
755	0.0041	–	805	0.0004	–	855	0.0000	–
756	1.6004	–	806	0.0106	–	856	0.0000	–
757	0.0001	–	807	0.0044	–	857	0.0000	–
758	0.0003	–	808	0.0008	–	858	0.0000	–
759	0.0001	–	809	0.0002	–	859	0.0000	–
760	0.0060	–	810	4.5901	–	860	0.0001	–
761	0.0012	–	811	0.0000	–	861	0.0003	–
762	0.0000	–	812	0.0001	–	862	0.0013	–
763	0.0000	–	813	1.0057	–	863	0.0012	–
764	0.0002	–	814	0.0007	–	864	0.0030	–
765	0.0000	–	815	0.0000	–	865	0.0021	–
766	0.0079	–	816	0.0002	–	866	0.0014	–
767	0.0009	–	817	0.0001	–	867	0.0012	–
768	0.0224	–	818	0.0001	–	868	0.0010	–
769	0.0007	–	819	0.0014	–	869	0.0005	–
770	0.0001	–	820	0.0002	–	870	0.0001	–
771	0.0000	–	821	0.0011	–	871	0.2757	–
772	0.0006	–	822	0.0693	–	872	2.1251	–
773	0.0000	–	823	3.5261	–	873	0.0000	–
774	0.0000	–	824	0.0053	–	874	0.0126	–
775	3.5000	–	825	0.0968	–	875	0.0000	–
776	0.0000	–	826	0.0004	–	876	0.0001	–
777	0.0032	–	827	0.0000	–	877	0.0001	–
778	0.0005	–	828	0.0000	–	878	0.0001	–
779	2.4898	–	829	0.0001	–	879	0.0004	–
780	0.0003	–	830	0.0000	–	880	0.0011	–
781	0.0002	–	831	0.2342	–	881	0.0037	–
782	0.0006	–	832	0.1212	–	882	0.0065	–
783	0.0010	–	833	0.0001	–	883	0.0218	–
784	0.2735	–	834	3.0886	–	884	0.0006	–
785	0.1667	–	835	0.0266	–	885	0.0015	–
786	0.4575	–	836	0.0003	–	886	0.0007	–
787	0.0001	–	837	0.0006	–	887	0.0011	–
788	0.4992	–	838	0.0014	–	888	0.0006	–
789	0.0011	–	839	1.8148	–	889	0.0008	–
790	0.0002	–	840	0.0008	–	890	0.0025	–
791	0.0003	–	841	0.0007	–	891	0.0017	–
792	0.0002	–	842	0.0017	–	892	0.0030	–
793	0.0014	–	843	0.0117	–	893	0.0172	–
794	2.2804	–	844	0.0007	–	894	0.0035	–
795	0.0010	–	845	0.0012	–	895	0.0025	–
796	5.0450	–	846	0.0007	–	896	0.0002	–
797	0.0009	–	847	0.0003	–	897	0.0002	–
798	0.0009	–	848	0.0021	–	898	0.2010	–
799	0.0006	–	849	0.0045	–	899	0.0000	–
800	0.0026	0.0023	850	0.0019	–	900	0.0024	0.0000

Table 35: Training and validation loss for the ASND model fine−tuned with 500 examples from step 901 to 1050.

Step	Tr. Loss	Val. Loss	Step	Tr. Loss	Val. Loss	Step	Tr. Loss	Val. Loss
901	0.0001	–	951	0.0005	–	1001	0.0012	–
902	2.4219	–	952	0.0000	–	1002	0.0014	–
903	0.0001	–	953	0.0111	–	1003	0.0007	–
904	0.0001	–	954	0.0002	–	1004	0.0006	–
905	0.0001	–	955	0.0000	–	1005	0.0003	–
906	0.0002	–	956	0.0001	–	1006	0.0002	–
907	1.6754	–	957	0.0001	–	1007	0.0000	–
908	0.0037	–	958	0.0001	–	1008	0.0001	–
909	0.3652	–	959	0.0006	–	1009	0.0001	–
910	2.7863	–	960	0.0053	–	1010	0.5619	–
911	0.0078	–	961	0.0171	–	1011	0.0001	–
912	0.0057	–	962	0.7524	–	1012	0.0001	–
913	0.0040	–	963	0.0213	–	1013	0.0003	–
914	0.0003	–	964	0.0074	–	1014	0.0004	–
915	1.2502	–	965	0.0004	–	1015	0.0002	–
916	0.0273	–	966	0.0026	–	1016	0.0029	–
917	0.0002	–	967	0.0013	–	1017	0.0021	–
918	0.0001	–	968	0.0003	–	1018	0.0029	–
919	0.0001	–	969	0.0002	–	1019	0.0023	–
920	0.0000	–	970	0.0001	–	1020	0.0065	–
921	0.0046	–	971	0.0000	–	1021	0.0012	–
922	0.0000	–	972	0.0000	–	1022	0.0002	–
923	2.3283	–	973	0.0000	–	1023	0.1225	–
924	0.0001	–	974	0.0002	–	1024	0.0002	–
925	0.0001	–	975	0.0001	–	1025	0.0002	–
926	0.0000	–	976	0.0000	–	1026	0.0003	–
927	0.9896	–	977	2.1206	–	1027	0.0006	–
928	2.3915	–	978	0.0001	–	1028	0.0006	–
929	0.0001	–	979	0.0001	–	1029	0.0019	–
930	0.0191	–	980	0.0003	–	1030	0.0011	–
931	0.0000	–	981	0.0007	–	1031	0.0016	–
932	0.0098	–	982	0.0005	–	1032	0.0015	–
933	2.1579	–	983	0.0008	–	1033	0.0005	–
934	0.0073	–	984	0.0014	–	1034	0.0001	–
935	0.2404	–	985	0.0016	–	1035	0.0000	–
936	0.0008	–	986	0.0011	–	1036	0.0000	–
937	0.0043	–	987	0.0001	–	1037	0.0000	–
938	0.0002	–	988	0.4787	–	1038	0.0000	–
939	0.0000	–	989	0.0002	–	1039	0.0001	–
940	0.0000	–	990	0.0819	–	1040	0.0000	–
941	0.0000	–	991	0.0001	–	1041	0.0000	–
942	0.0000	–	992	0.0001	–	1042	0.0000	–
943	0.0000	–	993	0.6307	–	1043	0.5629	–
944	0.0003	–	994	0.0000	–	1044	0.0000	–
945	0.0000	–	995	0.0001	–	1045	1.4632	–
946	0.0000	–	996	0.0001	–	1046	0.0017	–
947	1.1355	–	997	0.0003	–	1047	0.0002	–
948	0.0283	–	998	0.0001	–	1048	0.0001	–
949	0.0000	–	999	0.0001	–	1049	1.9563	–
950	0.0064	–	1000	0.0000	0.0011	1050	0.0004	–

Table 36: Training and validation loss for the ASND model fine−tuned with 500 examples from step 1051 to 1200.

Step	Tr. Loss	Val. Loss	Step	Tr. Loss	Val. Loss	Step	Tr. Loss	Val. Loss
1051	0.0010	–	1101	0.0002	–	1151	0.0001	–
1052	0.0010	–	1102	0.0003	–	1152	0.0001	–
1053	0.0011	–	1103	0.0010	–	1153	0.0006	–
1054	0.0026	–	1104	0.0002	–	1154	0.0013	–
1055	0.0018	–	1105	0.0003	–	1155	0.0001	–
1056	0.0018	–	1106	0.0006	–	1156	0.0010	–
1057	0.0060	–	1107	0.0026	–	1157	0.0002	–
1058	0.0010	–	1108	0.0017	–	1158	0.0002	–
1059	0.0006	–	1109	0.0012	–	1159	0.0000	–
1060	0.0003	–	1110	0.0033	–	1160	0.0001	–
1061	0.0008	–	1111	0.0035	–	1161	0.0004	–
1062	0.0001	–	1112	0.0057	–	1162	1.7521	–
1063	0.8599	–	1113	0.0024	–	1163	0.0013	–
1064	0.0000	–	1114	0.0017	–	1164	0.0000	–
1065	0.0000	–	1115	0.0005	–	1165	0.0001	–
1066	0.0002	–	1116	1.6416	–	1166	0.0002	–
1067	0.0000	–	1117	0.0002	–	1167	0.0000	–
1068	0.0000	–	1118	2.6173	–	1168	0.0003	–
1069	0.0001	–	1119	0.0001	–	1169	0.0002	–
1070	0.0001	–	1120	0.0910	–	1170	0.0001	–
1071	2.8282	–	1121	1.2988	–	1171	0.0001	–
1072	0.0011	–	1122	0.0004	–	1172	0.0002	–
1073	0.0003	–	1123	0.0000	–	1173	2.7522	–
1074	2.6358	–	1124	0.0001	–	1174	2.5839	–
1075	0.0005	–	1125	0.0001	–	1175	0.0002	–
1076	0.0009	–	1126	0.0000	–	1176	0.0004	–
1077	0.0009	–	1127	0.7807	–	1177	0.0004	–
1078	0.0004	–	1128	0.0000	–	1178	0.0009	–
1079	0.0003	–	1129	0.0000	–	1179	2.1099	–
1080	0.0003	–	1130	0.0000	–	1180	0.0010	–
1081	0.0001	–	1131	0.0000	–	1181	0.0004	–
1082	0.0001	–	1132	0.0000	–	1182	0.2743	–
1083	0.0075	–	1133	0.0000	–	1183	0.0003	–
1084	0.0001	–	1134	0.0000	–	1184	0.0003	–
1085	0.0001	–	1135	0.0017	–	1185	0.0007	–
1086	0.0009	–	1136	0.0000	–	1186	0.5136	–
1087	0.0001	–	1137	0.0000	–	1187	0.0004	–
1088	0.0016	–	1138	0.0001	–	1188	0.0007	–
1089	0.0005	–	1139	0.0001	–	1189	0.0335	–
1090	0.0000	–	1140	0.0001	–	1190	0.0041	–
1091	0.0001	–	1141	0.0008	–	1191	0.0008	–
1092	2.1060	–	1142	0.0005	–	1192	0.0011	–
1093	0.0013	–	1143	1.1600	–	1193	0.0004	–
1094	0.0002	–	1144	0.0001	–	1194	0.0044	–
1095	0.0006	–	1145	0.0004	–	1195	0.0002	–
1096	0.0001	–	1146	0.0006	–	1196	0.0002	–
1097	0.0015	–	1147	0.0004	–	1197	0.0585	–
1098	0.0001	–	1148	0.0037	–	1198	0.0002	–
1099	2.3416	–	1149	0.0014	–	1199	0.0003	–
1100	0.2799	0.0003	1150	0.0015	–	1200	0.0014	0.0001

Table 37: Training and validation loss for the ASND model fine−tuned with 500 examples from step 1201 to 1350.

Step	Tr. Loss	Val. Loss	Step	Tr. Loss	Val. Loss	Step	Tr. Loss	Val. Loss
1201	0.0001	–	1251	0.0002	–	1301	0.0010	–
1202	2.2892	–	1252	0.0001	–	1302	0.0001	–
1203	0.0003	–	1253	0.0002	–	1303	0.0004	–
1204	0.0001	–	1254	0.0010	–	1304	1.6010	–
1205	0.0002	–	1255	0.0002	–	1305	0.0003	–
1206	0.0001	–	1256	0.0020	–	1306	0.0001	–
1207	0.0001	–	1257	0.0004	–	1307	0.0001	–
1208	1.7412	–	1258	0.0007	–	1308	0.0001	–
1209	0.0001	–	1259	0.0008	–	1309	0.0021	–
1210	0.0001	–	1260	0.0010	–	1310	0.0003	–
1211	0.0002	–	1261	0.0022	–	1311	0.0002	–
1212	0.0029	–	1262	0.0007	–	1312	0.0003	–
1213	0.0100	–	1263	0.0039	–	1313	0.0004	–
1214	0.2137	–	1264	0.0035	–	1314	0.0008	–
1215	1.3653	–	1265	0.0005	–	1315	0.0002	–
1216	0.0014	–	1266	0.0005	–	1316	0.0006	–
1217	1.8687	–	1267	0.0004	–	1317	0.0007	–
1218	0.0010	–	1268	0.5816	–	1318	0.0007	–
1219	1.4685	–	1269	0.0002	–	1319	0.0009	–
1220	1.7901	–	1270	0.0004	–	1320	0.0007	–
1221	0.2557	–	1271	0.0005	–	1321	1.3971	–
1222	0.0052	–	1272	0.0005	–	1322	0.0003	–
1223	0.0058	–	1273	0.0008	–	1323	0.0015	–
1224	0.0040	–	1274	0.0009	–	1324	0.0005	–
1225	0.0018	–	1275	0.0011	–	1325	0.0003	–
1226	0.0003	–	1276	0.0091	–	1326	0.0008	–
1227	0.0006	–	1277	0.0041	–	1327	0.0004	–
1228	0.0009	–	1278	0.0007	–	1328	0.0005	–
1229	0.0004	–	1279	0.0002	–	1329	0.0009	–
1230	0.0012	–	1280	0.0002	–	1330	0.0033	–
1231	1.5761	–	1281	0.0003	–	1331	0.0005	–
1232	0.0002	–	1282	0.0001	–	1332	0.0005	–
1233	0.0005	–	1283	0.0009	–	1333	0.0005	–
1234	0.9249	–	1284	0.0002	–	1334	0.0003	–
1235	0.0006	–	1285	0.0003	–	1335	0.0004	–
1236	0.0005	–	1286	0.0007	–	1336	0.0004	–
1237	0.0005	–	1287	0.0003	–	1337	0.0001	–
1238	0.0008	–	1288	0.0002	–	1338	0.0008	–
1239	0.0011	–	1289	0.0004	–	1339	0.0011	–
1240	0.5673	–	1290	0.0003	–	1340	0.0021	–
1241	0.0015	–	1291	0.0005	–	1341	0.0010	–
1242	0.0021	–	1292	0.0001	–	1342	0.0015	–
1243	0.0010	–	1293	0.0335	–	1343	0.0035	–
1244	0.0006	–	1294	0.0003	–	1344	0.0054	–
1245	0.0006	–	1295	0.0014	–	1345	0.0030	–
1246	0.0005	–	1296	0.0010	–	1346	0.0023	–
1247	0.0003	–	1297	0.0021	–	1347	0.0016	–
1248	0.0001	–	1298	0.0033	–	1348	0.0004	–
1249	0.0001	–	1299	0.0007	–	1349	0.0005	–
1250	0.0002	–	1300	0.0013	0.0001	1350	0.0012	–

Table 38: Training and validation loss for the ASND model fine−tuned with 500 examples from step 1351 to 1500.

Step	Tr. Loss	Val. Loss	Step	Tr. Loss	Val. Loss	Step	Tr. Loss	Val. Loss
1351	0.0047	–	1401	0.0008	–	1451	0.0009	–
1352	0.0002	–	1402	0.0005	–	1452	0.0003	–
1353	0.0003	–	1403	0.0002	–	1453	0.0003	–
1354	0.0003	–	1404	0.0003	–	1454	0.0004	–
1355	0.0003	–	1405	0.0002	–	1455	0.0001	–
1356	0.1531	–	1406	0.0002	–	1456	0.0003	–
1357	0.0004	–	1407	0.0001	–	1457	0.0003	–
1358	0.0007	–	1408	0.0003	–	1458	0.0003	–
1359	0.0004	–	1409	0.0002	–	1459	0.0005	–
1360	0.0002	–	1410	0.0006	–	1460	0.0002	–
1361	0.0007	–	1411	0.0009	–	1461	0.0002	–
1362	0.0008	–	1412	0.0005	–	1462	0.0002	–
1363	0.0007	–	1413	0.0005	–	1463	0.6699	–
1364	0.0002	–	1414	0.0003	–	1464	0.0005	–
1365	0.0002	–	1415	0.0002	–	1465	1.1150	–
1366	0.0007	–	1416	0.0002	–	1466	0.0003	–
1367	0.0006	–	1417	3.2297	–	1467	0.0002	–
1368	0.0012	–	1418	0.0001	–	1468	0.0003	–
1369	0.0003	–	1419	0.0001	–	1469	0.0004	–
1370	0.0018	–	1420	0.0000	–	1470	0.0004	–
1371	0.0017	–	1421	0.0000	–	1471	0.0013	–
1372	0.0017	–	1422	0.0000	–	1472	0.0007	–
1373	0.0016	–	1423	0.0000	–	1473	0.0025	–
1374	0.0017	–	1424	0.0001	–	1474	0.0029	–
1375	0.0011	–	1425	0.0000	–	1475	0.0021	–
1376	0.0006	–	1426	0.0000	–	1476	0.0026	–
1377	0.1248	–	1427	0.0001	–	1477	0.0018	–
1378	0.0011	–	1428	0.0001	–	1478	1.8361	–
1379	0.0004	–	1429	0.0001	–	1479	0.0018	–
1380	0.0003	–	1430	0.0001	–	1480	2.7115	–
1381	0.0003	–	1431	0.0000	–	1481	0.0003	–
1382	0.0007	–	1432	0.0007	–	1482	0.0004	–
1383	0.0013	–	1433	0.0001	–	1483	0.0001	–
1384	0.0008	–	1434	0.0001	–	1484	0.0004	–
1385	0.0004	–	1435	0.0002	–	1485	0.0003	–
1386	0.0007	–	1436	0.0003	–	1486	1.5089	–
1387	0.0003	–	1437	0.0006	–	1487	0.0003	–
1388	0.0006	–	1438	0.0002	–	1488	0.0002	–
1389	0.0008	–	1439	0.0003	–	1489	0.3671	–
1390	0.0011	–	1440	0.0003	–	1490	0.0003	–
1391	0.0002	–	1441	0.0004	–	1491	0.0002	–
1392	0.0020	–	1442	0.0011	–	1492	0.0002	–
1393	0.0039	–	1443	0.0008	–	1493	0.0002	–
1394	0.0039	–	1444	0.0006	–	1494	0.0004	–
1395	0.0005	–	1445	0.0009	–	1495	0.0004	–
1396	2.5103	–	1446	0.0008	–	1496	0.0009	–
1397	0.0009	–	1447	0.0005	–	1497	0.0005	–
1398	0.0027	–	1448	1.0789	–	1498	0.0008	–
1399	0.0025	–	1449	0.0004	–	1499	0.0009	–
1400	0.0015	0.0004	1450	0.0005	–	1500	0.0015	0.0006
